# A partially fluorinated ligand for two super-hydrophobic porous coordination polymers with classic structures and increased porosities

**DOI:** 10.1093/nsr/nwaa094

**Published:** 2020-05-08

**Authors:** Chao Wang, Dong-Dong Zhou, You-Wei Gan, Xue-Wen Zhang, Zi-Ming Ye, Jie-Peng Zhang

**Affiliations:** MOE Key Laboratory of Bioinorganic and Synthetic Chemistry, School of Chemistry, Sun Yat-Sen University, Guangzhou 510275, China; MOE Key Laboratory of Bioinorganic and Synthetic Chemistry, School of Chemistry, Sun Yat-Sen University, Guangzhou 510275, China; MOE Key Laboratory of Bioinorganic and Synthetic Chemistry, School of Chemistry, Sun Yat-Sen University, Guangzhou 510275, China; MOE Key Laboratory of Bioinorganic and Synthetic Chemistry, School of Chemistry, Sun Yat-Sen University, Guangzhou 510275, China; MOE Key Laboratory of Bioinorganic and Synthetic Chemistry, School of Chemistry, Sun Yat-Sen University, Guangzhou 510275, China; MOE Key Laboratory of Bioinorganic and Synthetic Chemistry, School of Chemistry, Sun Yat-Sen University, Guangzhou 510275, China

**Keywords:** porous coordination polymers, metal-organic frameworks, super-hydrophobicity, flexibility

## Abstract

3-Ethyl-5-trifluoromethyl-1,2,4-triazole is synthesized by a one-pot reaction. Using this asymmetric triazole ligand bearing one trifluoromethyl and one ethyl as side groups, we construct two new porous coordination polymers, MAF-9 and MAF-2F, being isostructural with the classic hydrophobic and flexible materials, FMOF-1 and MAF-2, based on symmetric triazole ligands bearing two trifluoromethyl groups or two ethyl groups, respectively. MAF-9 and MAF-2F can adsorb large amounts of organic solvents but completely exclude water, showing superhydrophobicity with water contact angles of 152^o^ in between those of FMOF-1 and MAF-2. MAF-9 exhibits very large N_2_-induced breathing and colossal positive and negative thermal expansions like FMOF-1, but the lower molecular weight and smaller volume of MAF-9 give 16% and 4% higher gravimetric and volumetric N_2_ uptakes, respectively. In contrast, MAF-2F is quite rigid and does not show the inversed temperature-dependent N_2_ adsorption and large guest-induced expansion like MAF-2. Further, despite the higher molecular weight and larger volume, MAF-2F possesses 6% and 25% higher gravimetric and volumetric CO_2_ uptakes, respectively. These results can be explained by the different pore sizes and side group arrangements in the two classic framework prototypes, which demonstrate the delicate roles of ligand side groups in controlling porosity, surface characteristic and flexibility.

## Teaser

By partial replacement of the non-coordinative ligand side groups, the classic porous framework materials can be easily synthesized with their main adsorption properties retained or even improved.

## INTRODUCTION

As a new type of adsorbent showing high structural regularity and extremely rich structural diversity, porous coordination polymers (PCPs) or metal-organic frameworks (MOFs) have attracted great attention for achieving extraordinary properties [1–4]. PCPs can have not only much higher flexibility [5–12], but also much higher hydrophobicity than other types of adsorbents [13–34]. Introducing hydrophobic side groups on the organic ligand is the main strategy for synthesizing/designing hydrophobic PCPs [20–33]. Due to the extremely high electronegativity of fluorine, fluorinated organic compounds usually have high hydrophobicity [20–31]. PCPs constructed by perfluorinated organic ligands are of particular interest, but reported examples are very rare because fluorinated ligands are difficult to synthesize [23–30].

[Ag(bftz)] (FMOF-1, Hbftz = 3,5-bis(trifluoromethyl)-1,2,4-triazole) is a classic PCP constructed by a perfluorinated organic ligand [27], which can readily adsorb carbon dioxide and various hydrocarbons and completely exclude water [22,23]. FMOF-1 is also noteworthy for its remarkably large N_2_-induced framework breathing, colossal positive/negative thermal expansion, and low dielectric constant [28,35]. Nevertheless, the synthesis of the perfluorinated organic ligand Hbftz requires six-step reactions, and the synthesis of FMOF-1 also requires several reaction-evaporation-recrystallization steps using several organic solvents [27,36], which impede the study/application of this classic PCP.

Based on the more common alkyl groups, we have designed and synthesized a series of hydrophobic porous metal azolate frameworks (MAFs) with high stability and interesting properties [37]. For example, [Cu(detz)] (MAF-2, Hdetz = 3,5-diethyl-1,2,4-triazole), as a rare Cu(I)-based PCP showing high stability toward water and oxygen, can be used to separate organic solvents from water and sense oxygen in air and water [38–39]. Besides multimode distortion of the Cu(I)-triazolate scaffold in response to different organic molecules, MAF-2 also exhibits aperture dynamism originated from the flexible ethyl groups, which give inversed temperature-dependence of N_2_ adsorption [38].

Recently, we found that partially fluorinated azoles are relatively easy to synthesize and can be used to construct highly hydrophobic and stable PCPs [20,21]. For example, 3-methyl-5-trifluoromethyl-1,2,4-triazole can be synthesized from trifluoroacetohydrazide and acetamidine hydrochloride by a one-pot reaction [40]. Here, we report two new superhydrophobic PCPs, namely [Ag(fetz)] (MAF-9) and [Cu(fetz)] (MAF-2F), being isostructural with the classic materials FMOF-1 and MAF-2, respectively, by using an easily synthesized, partially fluorinated ligand 3-ethyl-5-trifluoromethyl-1,2,4-triazole (Hfetz).

## RESULTS AND DISCUSSION

### Synthesis

**Figure 1.**
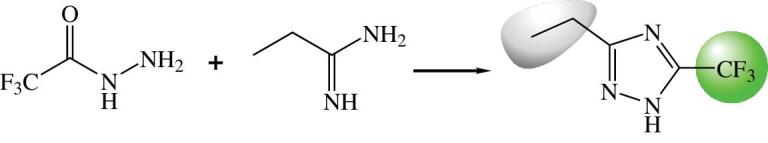
One-step synthesis of Hfetz. The sizes and shapes of the side groups are highlighted.

The ligand Hfetz can be synthesized in high yield by a one-pot reaction between trifluoroacetohydrazide and propionamidine hydrochloride (Fig. 1; Figs S1 and S2) [40]. Hfetz can dissolve in CHCl_3_, benzene, toluene, xylene, ethanol and methanol, but is insoluble in water. Room temperature diffusion of the methanol solution of Hfetz and aqueous solution of AgNO_3_ with toluene as a buffer layer yielded colorless, block-shaped single crystals of MAF-9. Solvothermal reaction of Cu(NO_3_)_2_ and Hfetz in water/toluene mixed solvent yielded single crystals of MAF-2F [37]. Microcrystalline MAF-9 and MAF-2F can be synthesized facilely by fast mixing of the toluene solution of Hfetz and the aqueous solution of AgNO_3_ at room temperature (Fig. S3), or by refluxing Cu_2_O nanocrystals and Hfetz in ethanol under oxygen-free conditions (Fig. S4).

Various Ag(I) 1,2,4-triazolate structures have been reported [41–45], but only one (3,5-diphenyl-1,2,4-triazolate, nonporous because of the bulky phenyl groups) is isostructural or isoreticular with FMOF-1 [41]. Ag(I) 3,5-diethyl-1,2,4-triazolate crystallizes as complicated three-dimensional (3D) coordination frameworks with inaccessible pores [42]. [Cu(dptz)] (Hdptz = 3,5-dipropyl-1,2,4-triazole) [37] and [Ag(diptz)]· C_6_H_6_ (Hdiptz = 3,5-diisopropyl-1,2,4-triazole) [42] possess the **nbo-a** typology of MAF-2, but they crystallize in the expanded cubic form and have negligible or no porosity because of the large side groups. We have also investigated the self-assembly of 3-methyl-5-trifluoromethyl-1,2,4-triazole and copper salts, but failed to obtain binary coordination polymers so far [20]. These results demonstrated the important role of uncoordinated side groups in determining the supramolecular structures [37].

### Structure

Single-crystal X-ray diffraction (SCXRD) revealed that MAF-9 and MAF-2F are isostructural with FMOF-1 (tetragonal *I*-42*d*) and MAF-2 (trigonal *R*-3), respectively (Table S1 and Figs S5 and S6). MAF-9 exhibits a unit-cell volume 1.9% smaller than that of FMOF-1, because the Ag−N bond lengths are ca. 0.1 Å shorter in the former structure (Table S2), which can be attributed to the electron withdrawing and donating nature of the ―CF_3_ and ―C_2_H_5_ groups which weakens and strengthens the coordination bond, respectively. The void ratio of MAF-9 (41.0%) is smaller than that of FMOF-1 (44.4%), but the crystallographic pore volume of MAF-9 is slightly larger than that of FMOF-1 (Table S3), because ―C_2_H_5_ is larger but lighter than ―CF_3_.

The unit-cell volume of MAF-2F is 3.0% larger than that of MAF-2, although the Cu−N bonds are only ca. 0.01 Å longer in the former structure (Tables S1 and S4). The variation of the unit-cell volume is mainly related to the conformation of the **nbo-a** network. The interplanar angles of the adjacent square nodes in MAF-2 and MAF-2F are 79.9^o^ and 84.4^o^, respectively (Fig. S7) [38]. An interplanar angle closer to 90^o^ means the network is closer to the ideal cubic symmetry with the largest volume. The relatively large interplanar angle of MAF-2F indicates that the ―CF_3_ groups in this structure prototype have larger steric hindrance effect than ―C_2_H_5_ groups. Although ―C_2_H_5_ is larger than ―CF_3_, this can happen when these side groups locate closely, since ―CF_3_ is larger than ―CH_2_―. The void ratio of MAF-2F (38.3%) is much larger than that of MAF-2 (31.3%), but the difference of the crystallographic pore volume is small (Table S5).

**Figure 2.**
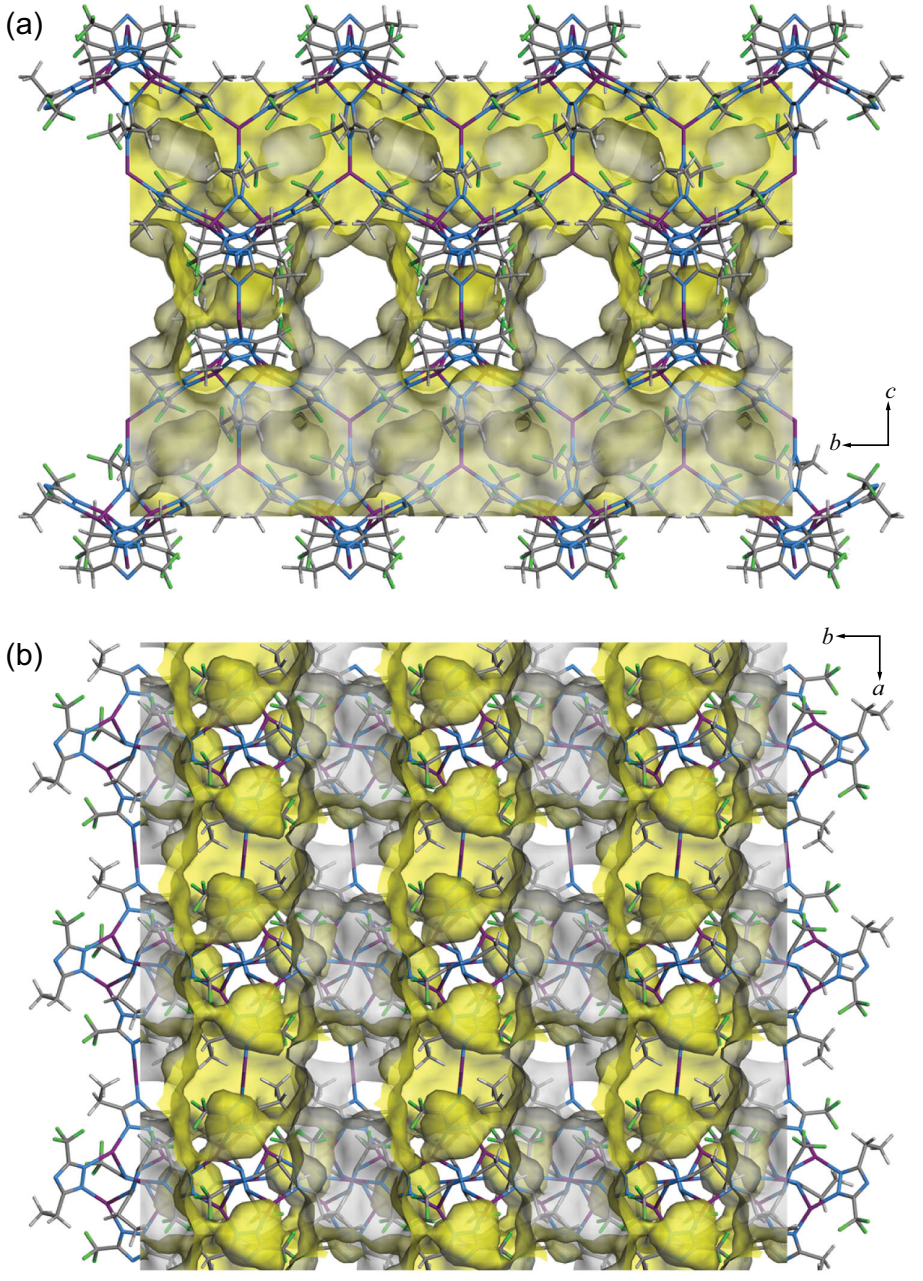
Framework and pore structures of MAF-9 viewing along (a) the *a*-axis and (b) the *c*-axis. The asymmetric fetz^–^ ligand is two-fold disordered in the crystal structure. Shown here is an average structure.

Similar to FMOF-1, MAF-9 possesses a 3D intersecting channel system with the 3-connected (10,3)-*b* (**ths**) topology (38.0%), and some very small, discrete cavities (3.0%) separated from the main 3D channel by the side groups (Fig. 2). Because some of the ―CF_3_ groups are replaced by ―C_2_H_5_ groups, some discrete cavities may become accessible from the 3D channel (Figs S8–S10). The channel apertures of MAF-9 and FMOF-1 viewing along the *a*- and *b*-axes are ellipsoidal (4.0−5.9 × 6.6 Å^2^) and rectangular (5.8 × 7.9 Å^2^), respectively, because the long and flexible ethyl groups locate at the aperture corners.

**Figure 3.**
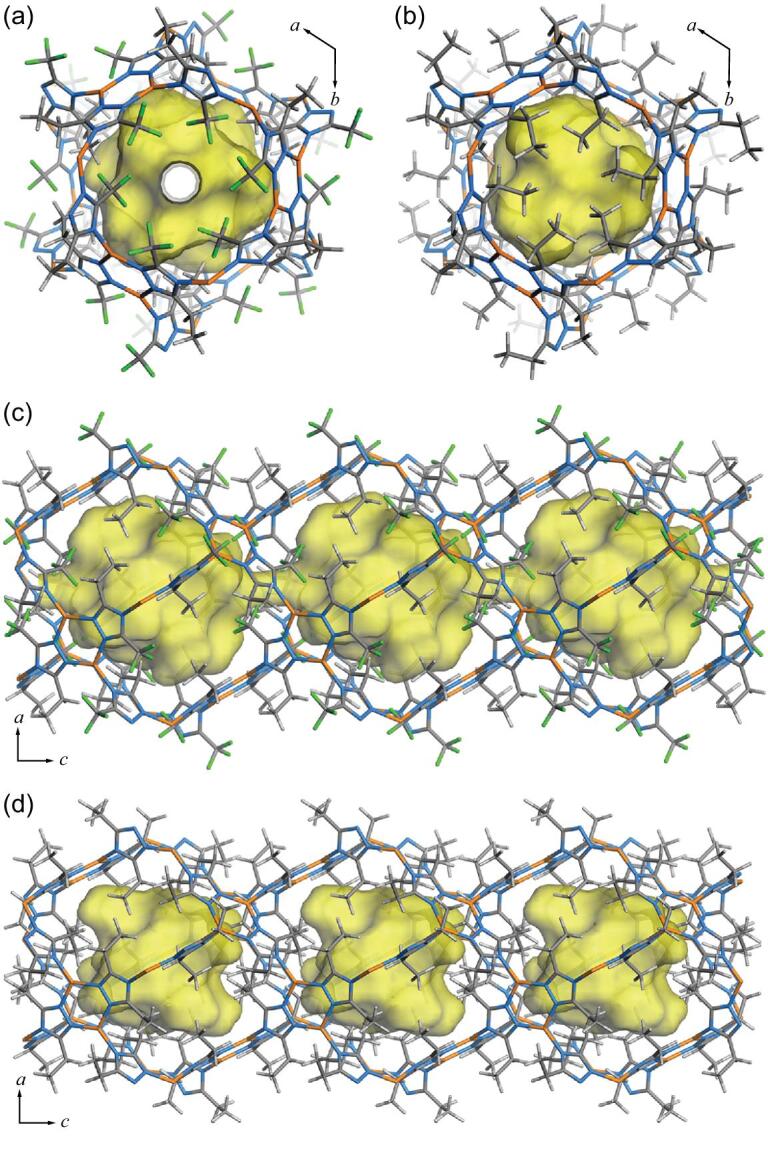
Framework and pore structures of (a, c) MAF-2F and (b, d) MAF-2 viewing along the *c*-axis (a, b) and the *a*-axis (c, d).

Just like MAF-2 possessing a distorted **nbo-a** coordination network and a distorted **bcu** pore system, there are large cavities and two types of apertures in MAF-2F. Along the *c*-axis, the aperture with an effective diameter of 2.5 Å is defined by six surrounding ―CF_3_ groups (Fig. 3a), which is much larger than that of MAF-2 (1.1 Å) defined by six ―C_2_H_5_ groups (Fig. 3b). Another type of aperture (not pointing to special crystallographic direction) is surrounded by four ―C_2_H_5_ groups and two ―CF_3_ groups with negligible effective size (Fig. 3c), being similar with that of MAF-2 (Fig. 3d). In other words, in the static point of view, the pore systems of MAF-2 and MAF-2F can be regarded as 0D and 1D, respectively, for a guest molecule (e.g. H_2_) with a diameter smaller than 2.5 Å (Fig. 3).

### Stability and hydrophobicity

**Figure 4.**
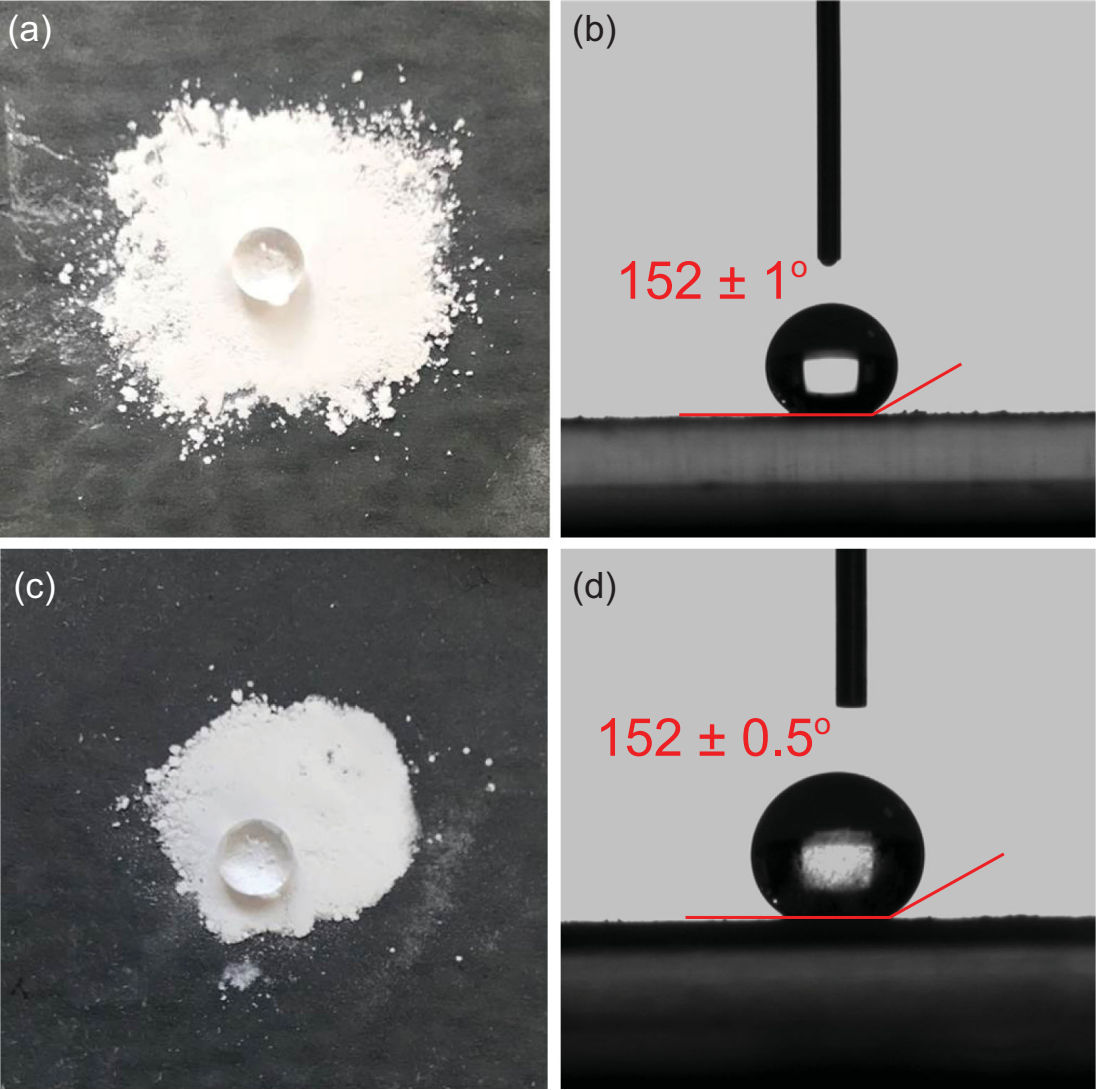
Typical photographs for water contact angle tests for (a, b) MAF-9 and (c, d) MAF-2F.

Thermogravimetry showed long plateaus from room temperature to 280°C for MAF-9 and MAF-2F, meaning that the as-synthesized samples contained no guest molecules (Figs S11 and S12), exemplifying their hydrophobic pores. Compacted samples of microcrystalline MAF-9 and MAF-2F both show water contact angles of 152^o^ (Fig. 4) and glide angles of less than 4^o^ (Figs S13 and S14), meaning that their crystal surfaces are superhydrophobic, which have only been observed in a few PCPs [13–16,21–24,33,34]. The water contact angle of FMOF-1 was reported as 158^o^ [22], while that of MAF-2 was measured as 140^o^ (Fig. S15), which exemplifies the higher hydrophobicity of ―CF_3_ compared with ―C_2_H_5_, and the ability of tuning hydrophobicity by mixing these functional groups.

MAF-9 can keep its powder X-ray diffraction (PXRD) pattern and color unchanged in water and/or under sunlight at room temperature for at least one year (Fig. S3). MAF-2F can also maintain its PXRD pattern and color in water and/or humid air at room temperature for at least three months (Fig. S4). For comparison, FMOF-1 was reported to be stable after being exposed to saturated water vapor for 70 days at room temperature [28]. MAF-2 was reported to be able to keep its PXRD pattern unchanged in water for at least one year, but turned light green in humid conditions after several days due to the oxidation of the crystal surface [39].

### Gas adsorption and flexibility

**Figure 5.**
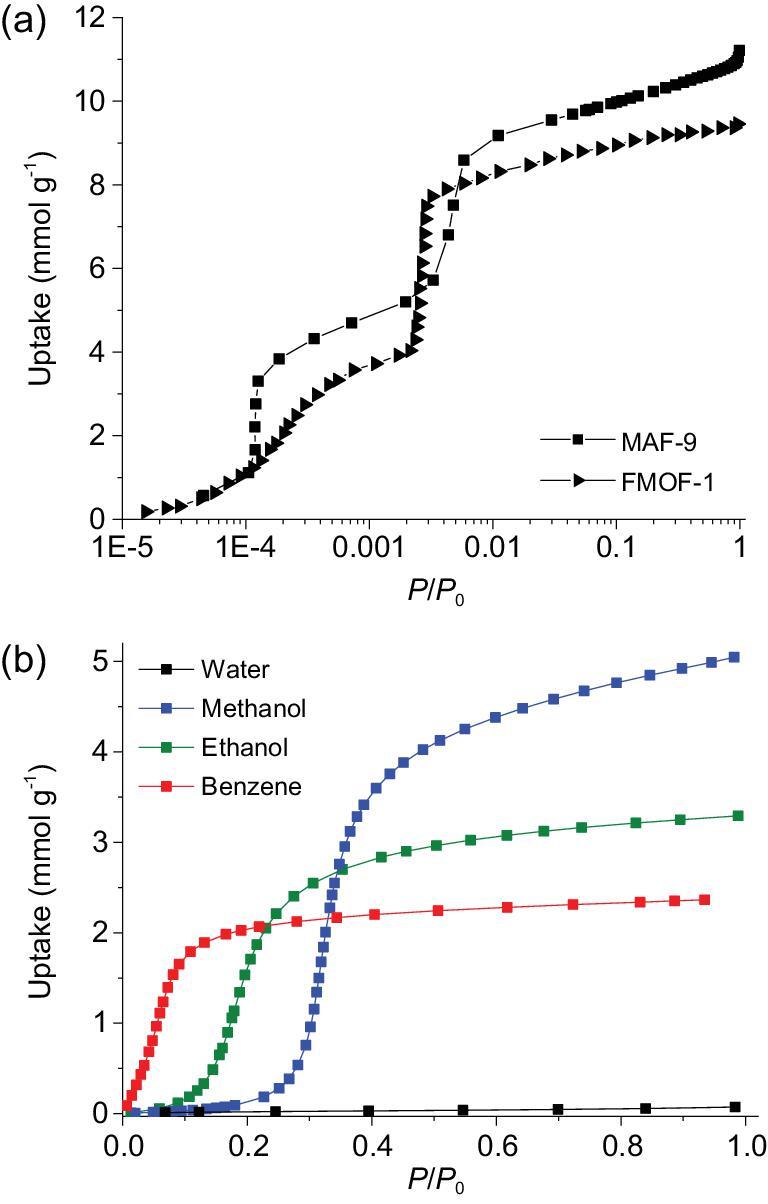
(a) 77-K N_2_ adsorption isotherms of MAF-9 and FMOF-1 and (b) 298-K water, methanol, ethanol and benzene vapor adsorption isotherms of MAF-9.

MAF-9 shows an apparent type-I N_2_ adsorption isotherm at 77 K, but there is an additional step around *P*/*P*_0_ = 0.001 (Fig. 5a and Fig. S16), which is very similar with that of FMOF-1 [27]. The saturated N_2_ uptake and corresponding experimental pore volume of MAF-9 are 16% larger than those of FMOF-1, consistent with the difference of their molecular weights (Table S3). Actually, the two isostructural PCPs show similar or the same host–guest stoichiometries of N_2_/Ag ≈ 1.3 and N_2_/Ag = 3.0 at the two isotherm steps (Fig. S16). The very similar N_2_ adsorption isotherms imply that they share the same adsorption mechanism, which has been elucidated by *in situ* SCXRD for FMOF-1, i.e. the host first contracts (–8.6%) by adsorbing N_2_ only in the 3D channel, and then expands to a state larger than the guest-free state (3.4%) by additional adsorption of N_2_ not only in the 3D channel but also in the 0D cavities [35]. The higher host–guest stoichiometry of MAF-9 at the first isotherm step might be attributed to the existence of some accessible 0D cavities (Figs S8–S10).

Considering that guest-free FMOF-1 is also highly flexible toward temperature, we measured the SCXRD structure of MAF-9 at low temperature (Table S1), giving very large positive and negative thermal expansion coefficients (α*_a_* = 2.38 × 10^−4^ K^−1^, α*_c_* = −2.06 × 10^−4^ K^−1^ and *β* = 2.68 × 10^−4^ K^−1^), being similar with those reported for FMOF-1 under vacuum (α*_a_* = 2.3 × 10^−4^ K^−1^, α*_c_* = −1.7 × 10^−4^ K^−1^ and *β* = 3.0 × 10^−4^ K^−1^) [35]. Note that, because MAF-9 has a smaller unit-cell volume, its volumetric porosity is also higher than that of FMOF-1 (Table S3).

**Figure 6.**
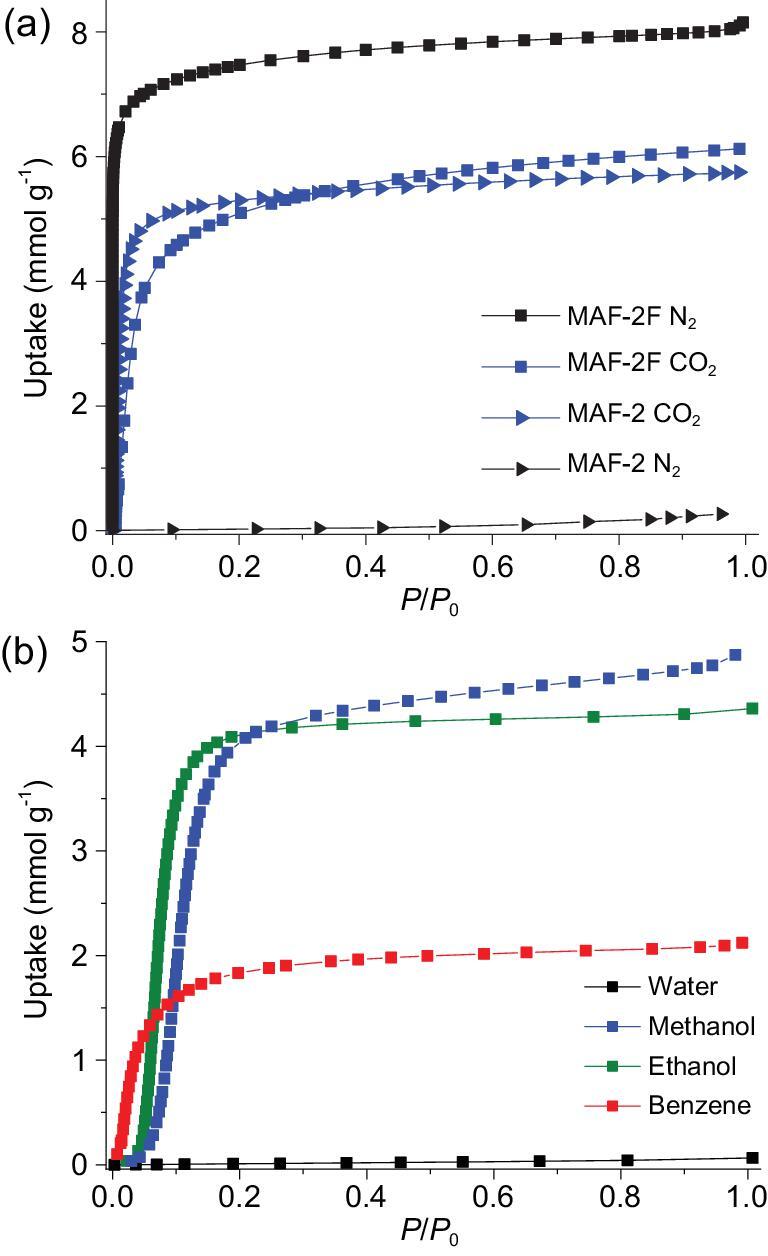
(a) 77-K N_2_ and 195-K CO_2_ adsorption isotherms of MAF-2F and MAF-2 and (b) 298-K water, methanol, ethanol and benzene vapor adsorption isotherms of MAF-2F.

MAF-2F exhibits a typical type-I N_2_ adsorption isotherm at 77 K (Fig. 6a; Figs S17 and S18). The pore volume calculated from the N_2_ isotherm fits well with crystallographic value (Table S5). In contrast, MAF-2 cannot adsorb N_2_ at 77 K, because the static sizes of the apertures are too small and the ―C_2_H_5_ groups are not dynamic enough at such a low temperature [38]. In this context, the relatively large aperture of MAF-2F along the *c*-axis and the dynamism of ―CF_3_ groups should be responsible for its N_2_ adsorption at 77 K, since the static size of the aperture is just slightly smaller than the guest molecule. At 195 K, MAF-2F shows a type-I CO_2_ isotherm with a pore volume slightly smaller than the crystallographic value (Table S5). Regardless of its higher molecular weight, the gravimetric saturated CO_2_ uptake of MAF-2F is 6% higher than that of MAF-2 [46]. In the volumetric point of view, the saturated CO_2_ uptake of MAF-2F is 25% higher than that of MAF-2. More straightforwardly, the host–guest stoichiometry of MAF-2F (1.39 CO_2_/Cu) is significantly larger than that of MAF-2 (1.08 CO_2_/Cu), meaning that the CO_2_ molecules arrange differently in the two isostructural PCPs. The different CO_2_ adsorption mechanisms of MAF-2F and MAF-2 can be visualized by Grand Canonical Monte Carlo (GCMC) simulations (Figs S19 and S20).

### Vapor adsorption and hydrophobicity/flexibility

MAF-9 shows type-V methanol, ethanol and benzene vapor adsorption isotherms, meaning that the host–guest interactions are weaker than the guest–guest interactions (Fig. 5b and Fig. S21) [47]. The initial isotherm slope follows benzene > ethanol > methanol, consistent with the trends of guest hydrophobicity and molecular weight, which can be explained by the fact that a larger molecule generally has stronger interaction with the host framework. The benzene adsorption capacity of MAF-9 (2.36 mmol g^−1^) is 6% higher than that of FMOF-1 (2.23 mmol g^−1^) [23]. Thermogravimetry showed that MAF-9 can also adsorb considerable amounts of large aromatic molecules such as *p*-xylene, *m*-xylene, *o*-xylene, cyclohexane and mesitylene (Fig. S11). By contrast, MAF-9 completely excludes water (0.07 mmol g^−1^ at *P*/*P*_0_ = 0.99), highlighting its high hydrophobicity (Fig. 5b). PXRD showed that the unit-cell parameters of MAF-9 in water are almost the same as those in air (Figs S22–S33 and Table S6), consistent with its high hydrophobicity. On the other hand, in organic solvents, the unit-cell volume of MAF-9 can increase up to 6.1% (*o*-xylene) or decrease up to 0.7% (ethanol); the *a*-axis can increase up to 5.3% (*o*-xylene) or decrease up to 1.5% (ethanol); and the *c*-axis can increase up to 2.2% (ethanol) or decrease up to 4.8% (mesitylene) (Table S6).

MAF-2F also shows type-V adsorption isotherms for methanol, ethanol and benzene, and completely excludes water (0.06 mmol g^−1^ at *P*/*P*_0_ = 0.99), being similar with MAF-9, FMOF-1 and MAF-2 (Fig. 6b and Fig. S34) [23,38]. Using the saturated methanol, ethanol and benzene uptakes of 4.8, 4.4 and 2.1 mmol g^−1^, the host–guest stoichiometries can be calculated as 1.09, 1.00 and 0.48 guest/Cu, respectively, just the same as those of MAF-2, meaning that these relatively large guest molecules have the same and ordered arrangements in the two analogues [38].

PXRD showed that MAF-2F shows negligible volume change (Δ*V* < 0.5%) after adsorbing water, methanol, ethanol or benzene (Figs S35–S40 and Table S7). In contrast, MAF-2 expands 4.4% and transforms from the trigonal conformation to the cubic conformation after adsorbing benzene [38]. This indicates that MAF-2F is much less flexible than MAF-2. As exemplified by the larger interplanar angle of MAF-2F, the six ―CF_3_ groups gathering at the apertures running along the *c*-axis endure stronger steric hindrance with each other, which can prevent the Cu(I) triazolate framework from guest-induced distortion. It should be noted that, even if the Cu(I) triazolate framework of MAF-2F expands to adopt the cubic symmetry, the presence of two types of apertures (surrounded by different numbers of ―CF_3_ and ―C_2_H_5_ groups) in a 1:3 ratio gives the whole framework a trigonal symmetry.

## CONCLUSION

By mixing the trifluoromethyl and ethyl groups in the triazolate ligand, we obtained two new PCPs being isostructual with the classic hydrophobic and flexible PCPs based on symmetric triazole ligands either fully fluorinated or non-fluorinated. The new PCPs exhibit superhydrophobicity in between the fully-fluorinated and non-fluorinated PCPs, but the new ligand and new PCPs are much easier to synthesize. Interestingly, regardless of changing the trifluoromethyl group to ethyl group or changing the ethyl group to trifluoromethyl group, the new PCPs show higher gas adsorption capactites, which highlights the important role of trivial modification of the size, length and thickness of ligand side groups in PCPs with small pore sizes.

## METHODS

### Materials and measurements

All reagents and solvents were commercially available and used as received without further purification. Elemental analyses (EA) were performed with a Vario El elemental analyzer. Thermogravimetry analyses were performed using a TA Q50 instrument with a heating rate of 10.0°C/min under nitrogen. Water contact angles and slide angles were measured using the KRUSS DSA100 contact angle meter using compressed powders. Nuclear magnetic resonance spectrum was measured on an AVANCE III 400 MHz spectrometer. Mass spectrum was obtained by LTQ Orbitrap Elite LC/MS (ESI) equipment with MeOH as the mobile phase.

### Synthesis of Hfetz

The synthesis method reported for Hfmtz was used [40]. A mixture of ethyl trifluoroacetate (7.1 g, 50 mmol), hydyrazine monohydrate (2.0 g, 50 mmol) and tetrahydrofuran (250 mL) was stirred for 1 h at reflux temperature and then cooled to room temperature. After the addition of propionamidine hydrochloride (6.0 g, 55 mmol) and NaOH (2.2 g, 55 mmol), the resultant mixture was stirred for another 3 h at reflux temperature. The mixture was quenched with a cold saturated NaHCO_3_ solution (2.5 L) and extracted with ethyl acetate (500 mL × 3). The extracts were dried with Na_2_SO_4_, filtered, and concentrated under vacuum. The residue was sublimated to give white solid (7.26 g, 88% yield): *R_f_* 0.55 (hexane/ethyl acetate 4:1); m.p. 130.5–131.2 °C; ^1^H NMR (400 MHz, CD_3_OD) δ 2.86 (q, *J* = 7.7 Hz, 2H), 1.36 (t, *J* = 7.7 Hz, 3H); ESI-MS *m*/*z* Calcd. for C_5_F_3_H_5_N_3_^–^ [M−H]^–^: 164.04, found: 164.12. EA calcd for C_5_F_3_H_6_N_3_ (%): C, 36.37; N, 25.45; H, 3.66. Found. C, 36.90; N, 25.52; H, 3.62.

### Synthesis of [Ag(fetz)] (MAF-9)

Single crystals: toluene (2.5 mL) and a solution of Hfetz (0.0136 g, 0.08 mmol) in methanol (2.0 mL) were sequentially layered onto a solution of AgNO_3_ (0.0132 g, 0.08 mmol) in water (2.0 mL). After about two weeks, colorless crystals were collected for single-crystal X-ray diffraction analyses. Microcrystalline powders: a solution of Hfetz (0.165 g, 1 mmol) in toluene (20 mL) was poured into an aqueous solution (20 mL) of AgNO_3_ (0.170 g, 1 mmol). After the suspension was stirred for 2 h at room temperature, the white crystalline powder was filtered and washed by methanol (40 mL) three times, and then dried in air for 2 h (0.177 g, 65% yield). EA calcd(%) for AgC_5_F_3_H_5_N_3_: C 22.08, N 15.45, H 1.85; found: C 22.34, N 15.26, H 1.77.

### Synthesis of [Cu(fetz)] (MAF-2F)

Single crystals: a solution of Cu(NO_3_)_2_ · 3H_2_O (0.5 mmol, 120.8 mg) in water (3 mL) and a solution of Hfetz (0.5 mmol, 0.0825 g) in toluene (3 mL) were mixed and sealed in a 15-mL Teflon-lined reactor, heated at 160°C for 72 h, and then slowly cooled to room temperature to give colorless crystals. Microcrystalline powders: a solution of Hfetz (0.165 g, 1 mmol) in ethanol (10 mL) was added into a suspension of Cu_2_O nanoparticle (0.5 mmol) in ethanol (10 mL). N_2_ was bubbled into the mixture for 2 min to evacuate O_2_. The solution was sealed in a glass bottle, refluxed for 30 min, and then slowly cooled to room temperature. The resultant white crystalline powders were filtrated, washed with ethanol three times, and then dried in air for 2 h (0.218 g, 96% yield). EA calcd(%) for C_5_CuF_3_H_5_N_3_: C 26.38, N 18.46, H 2.21; found: C 26.81, N 18.40, H 2.37.

### Sorption measurements

Gas sorption isotherms of FMOF-1 and MAF-2 were adopted from the literature [27,46]. Gas and vapor sorption isotherms of MAF-9 and MAF-2F were measured with automatic volumetric adsorption apparatuses (ASAP 2020M or BELSORP-max). The measurement temperature was controlled by a liquid-nitrogen bath (77 K), a dry ice-acetone bath (195 K) or a water bath (298 K). Before the sorption experiments, the sample was treated in high vacuum for 2 h at 383 K. Experimental pore volume was calculated based on the saturated gas uptake (read at *P*/*P*_0_ = 0.95), using the liquid N_2_ density of 0.804 g cm^−3^ or liquid CO_2_ density of 1.104 g cm^−3^. Volumetric uptake was converted from the gravimetric uptake using the crystal density, supposing that the material did not change volume after adsorption, which was basically valid for MAF-2/MAF-2F during N_2_/CO_2_ adsorption. FMOF-1/MAF-9 breathed significantly during N_2_ adsorption with very similar amplitudes, so that the absolute volumetric uptakes have relatively large errors, but they can be compared with each other.

### X-ray crystallography

Single-crystal X-ray diffraction intensities of MAF-9, MAF-2F and MAF-2 were collected on a Pilatus XtaLAB P300DS or a Rigaku Oxford SuperNova single-crystal diffractometer by using graphite monochromated Cu-Kα radiation. Absorption corrections were applied by using the multi-scan program REQAB or CrysAlisPro. The structures were solved by the direct method and refined by the full-matrix least-squares method on *F*^2^ with SHELXTL-2014 package. Anisotropic thermal parameters were applied to all non-hydrogen atoms. The hydrogen atoms were generated geometrically. To keep the anisotropic thermal parameters of the disordered trifluoromethyl and ethyl groups of MAF-9 within reasonable limits, ISOR restrictions were used in the refinements. Crystal data were summarized in Table S1.

Description/analysis of the crystal structures used the true C−H bond length of 1.1 Å. The void ratio was calculated by the SOLV route of PLATON 130220, using the default setting (probe radius of 1.2 Å). The void ratio of MAF-9 containing disordered ethyl groups was calculated as the average value of the two values supposing the material adopts two extreme structures.

PXRD patterns were collected on a Bruker D8 DAVANCI X-ray powder diffractometer with CuK-α radiation in the transmission mode at room temperature. Pawley refinements of PXRD data were performed in the 2*θ* range of 5−40° on unit-cell parameters, zero point and background terms with Pseudo-Voigt profile function and Berar-Baldinozzi asymmetry correction function. All the indexing and refinements were performed by the Reflex plus module of Materials Studio 5.5.

### Computational details

All simulations/calculations were performed using the Materials Studio 5.5 package. All the gas adsorption sites were generated from Grand Canonical Monte Carlo (GCMC) simulations with the fixed pressure task (at 195 K and 1 atm) in the Sorption module. The host frameworks and CO_2_ molecules were both regarded as rigid. The simulation box contained one unit cell, and the Metropolis method based on the universal force field (UFF) was used. Mulliken charges calculated from Density Functional Theory (DFT) were adopted for all the atoms of the host frameworks and CO_2_ molecules [48], with the grid interval of 0.4 Å. The cutoff radius was chosen as 12.5 Å for the Lennard-Jones potential, and the electrostatic interactions and van der Waals interactions were handled using the Ewald and Atom based summation methods, respectively. All the equilibration steps and production steps were set as 5 × 10^6^.

Before the GCMC simulations, full geometry optimizations were performed according to the literature [21]. The widely used generalized gradient approximation (GGA) with the Perdew–Burke–Ernzerhof (PBE) functional and the double numerical plus d-functions (DND) basis set, as well as the DFT Semicore Pseudopotentials (DSPP) were used. The energy, gradient and displacement convergence criteria were set as 2 × 10^−5^ Ha, 4 × 10^−3^ Å and 5 × 10^−3^ Å, respectively.

## Supplementary Material

nwaa094_Supplemental_Files
