# Supplementary material for: A partially fluorinated ligand for two super-hydrophobic porous coordination polymers with classic structures and increased porosities
Source: Natl Sci Rev. 2020 May 8;8(3):nwaa094. doi: 10.1093/nsr/nwaa094 (PMC8288338; doi:10.1093/nsr/nwaa094)
Supplement: nwaa094_Supplemental_Files [file nwaa094_supplemental_files.zip › Supporting information.pdf]

Supporting Information for

A partially fluorinated ligand for two super-hydrophobic porous coordination polymers with classic structures and increased porosities

Chao Wang, Dong-Dong Zhou, You-Wei Gan, Xue-Wen Zhang, Zi-Ming Ye, and Jie-Peng Zhang*

MOE Key Laboratory of Bioinorganic and Synthetic Chemistry, School of Chemistry, Sun Yat-Sen University, Guangzhou 510275, China

Email: zhangjp7@mail.sysu.edu.cn

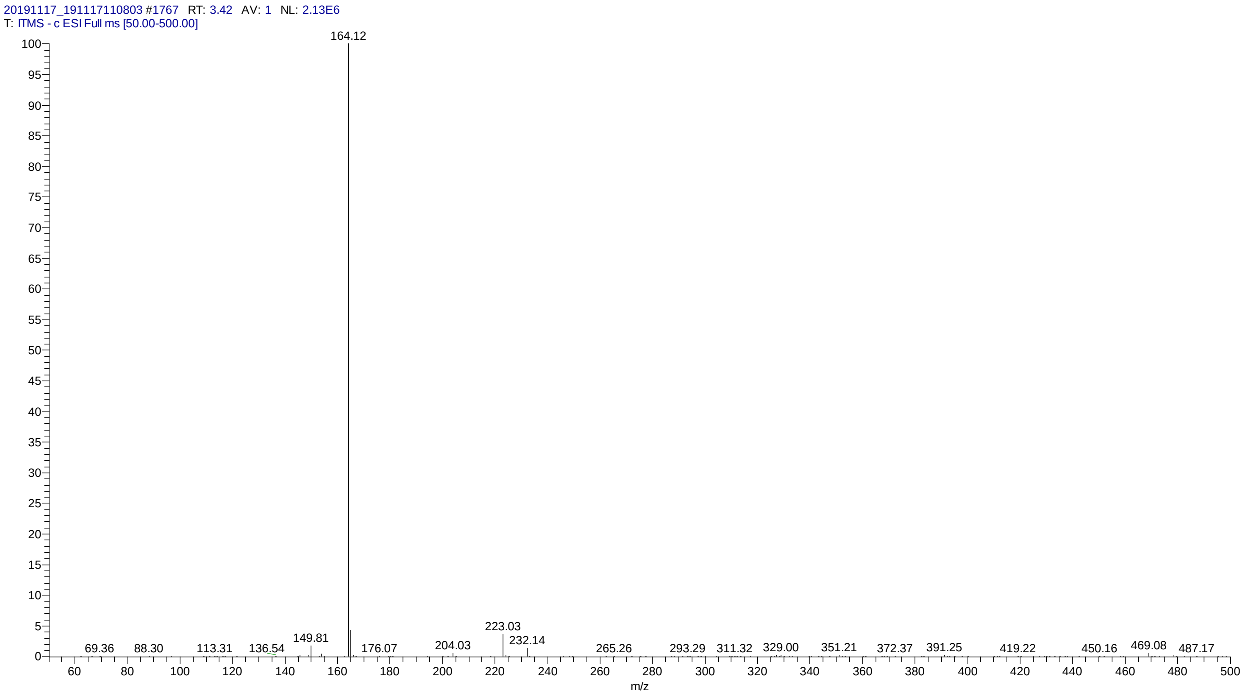

Figure S1. Mass spectrum of Hfetz.

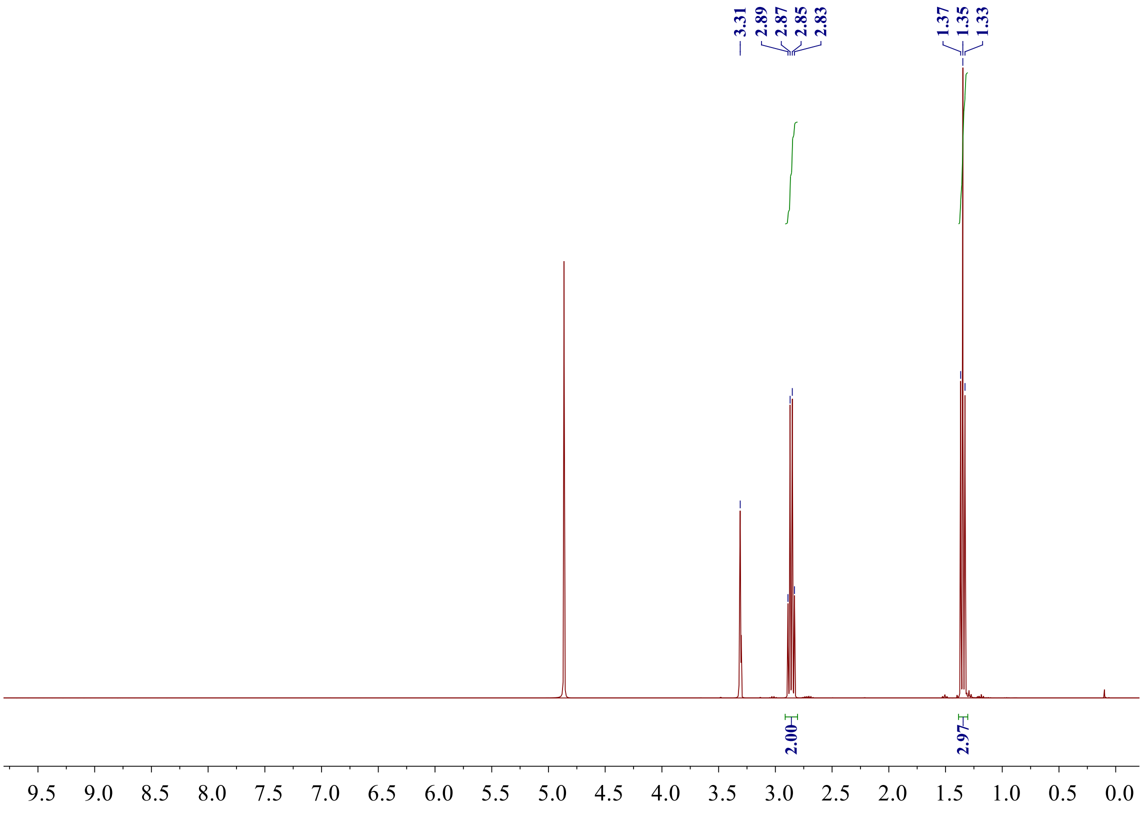

Figure S2. ^{1}H-nuclear magnetic resonance spectrum (400 MHz, CD_3OD, 298 K) of Hfetz.

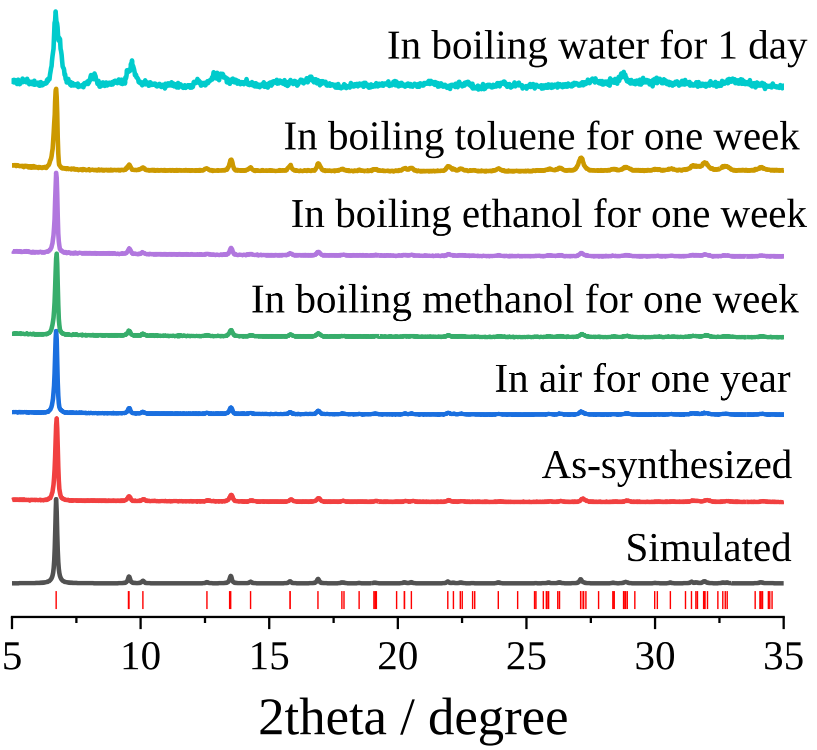

Figure S3. PXRD patterns of MAF-9 after different chemical treatments.

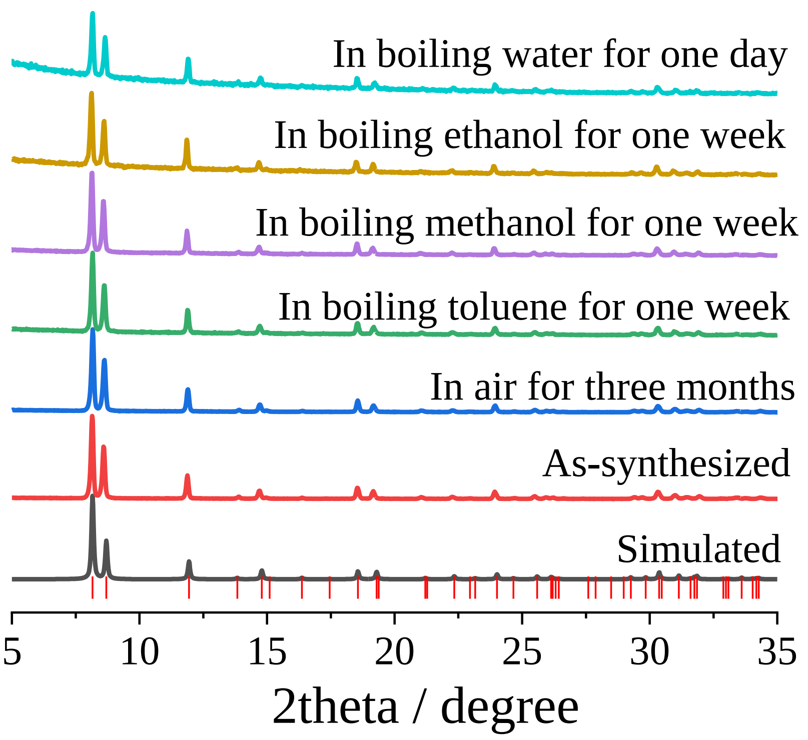

Figure S4. PXRD patterns of MAF-2F after different chemical treatments.

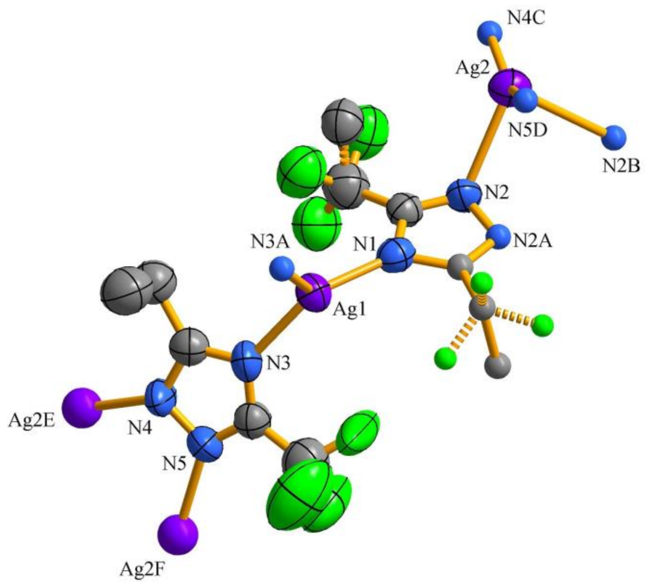

Figure S5. The coordination environment of MAF-9. Atoms in an asymmetric unit are drawn with thermal ellipsoids (probability at 25%). Hydrogen atoms are omitted for clarity. Symmetry codes: A = –*x*, 1−*y*, *z*; B = 1/2−*y*, 1/2+*x*, 1/2−*z*; C = −1+*y*, 1/2+*x*, 1/4+*z*; D = −*x*, −1/2+*y*, 1/4−*z*; E = −1/2+*y*, 1+*x*, −1/4+z; F: −*x*, 1/2+*y*, 1/4−*z*.

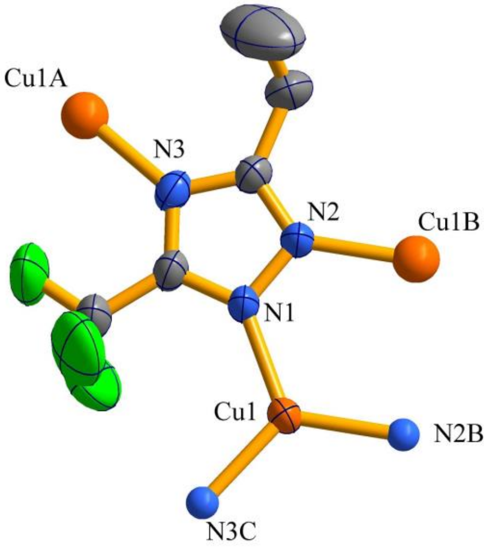

Figure S6. The coordination environment in MAF-2F. Atoms in an asymmetric unit are drawn with thermal ellipsoids (probability at 50%). Symmetry codes: A = 2/3+*x*−*y*, 1/3+*x*, 4/3−*z*; B = 1−*x*, 1−*y*, 1−z, C = −1/3+*y*, 1/3−*x*+*y*, 4/3−*z*. Hydrogen atoms are omitted for clarity.

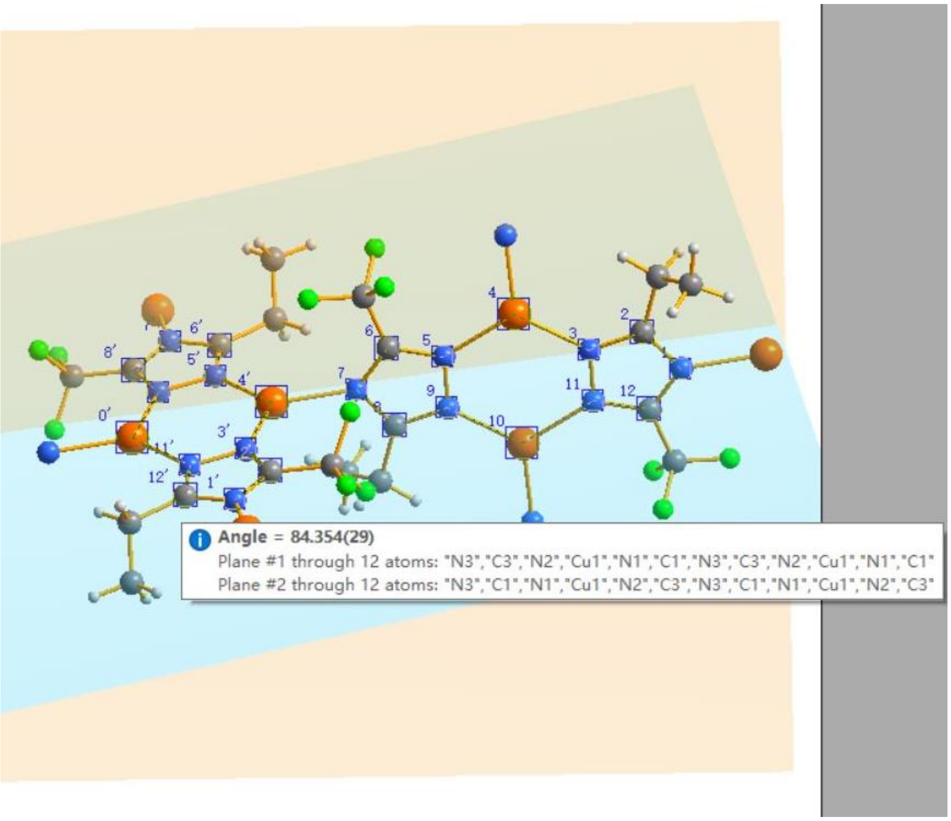

(a)

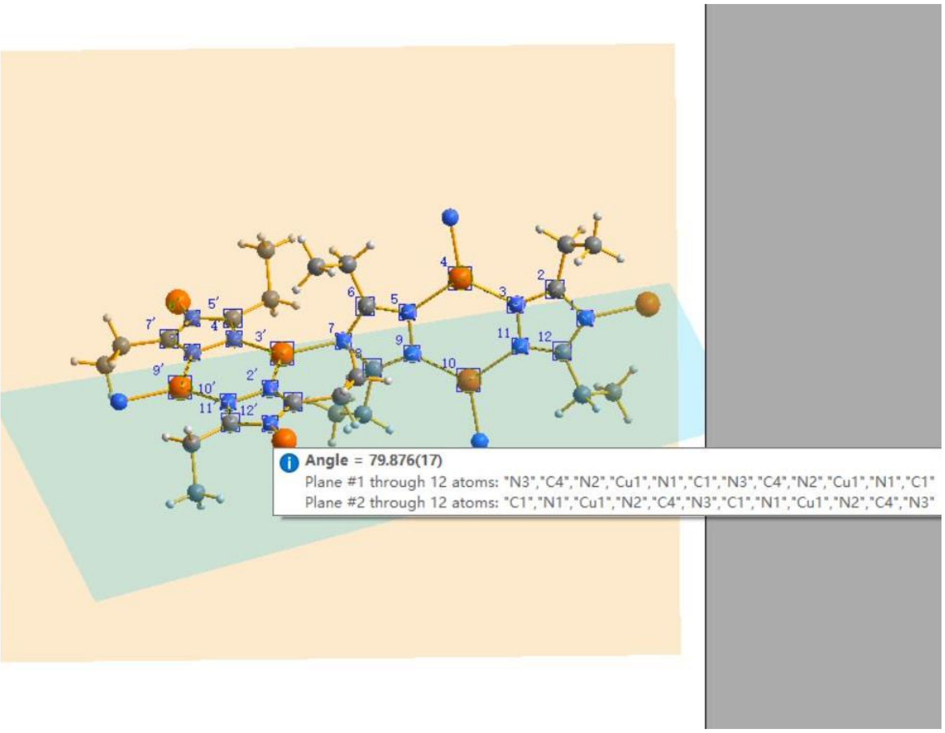

(b)

Figure S7. Dihedral angles of adjacent $Cu_2(tz)_2$ units in (a) MAF-2F and (b) MAF-2.

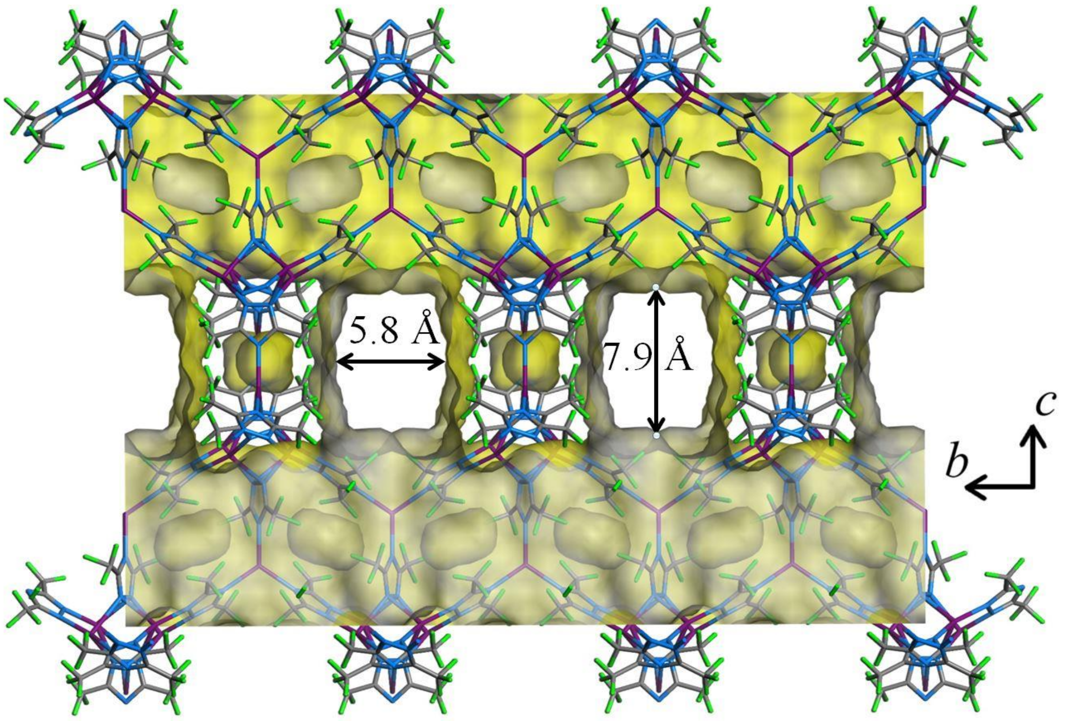

(a)

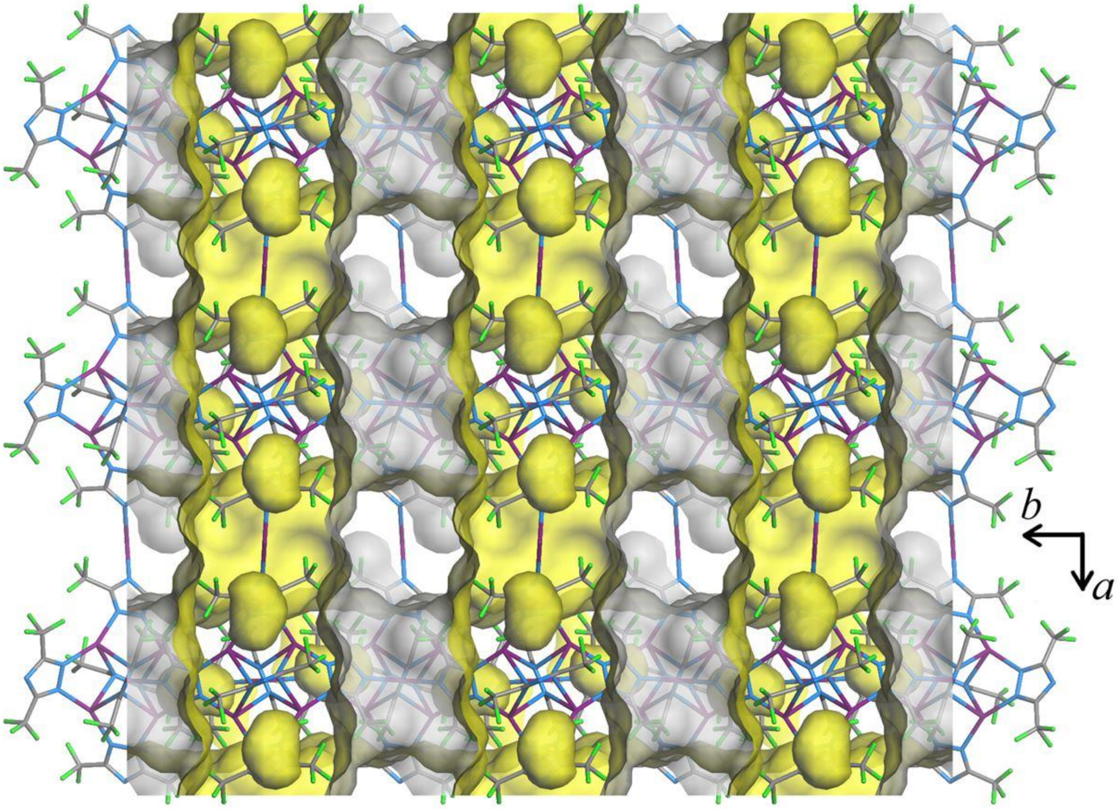

(b)

Figure S8. Framework and pore surface structures of FMOF-1 viewing along the (a) *a*-axis and (b) the *c*-axis.

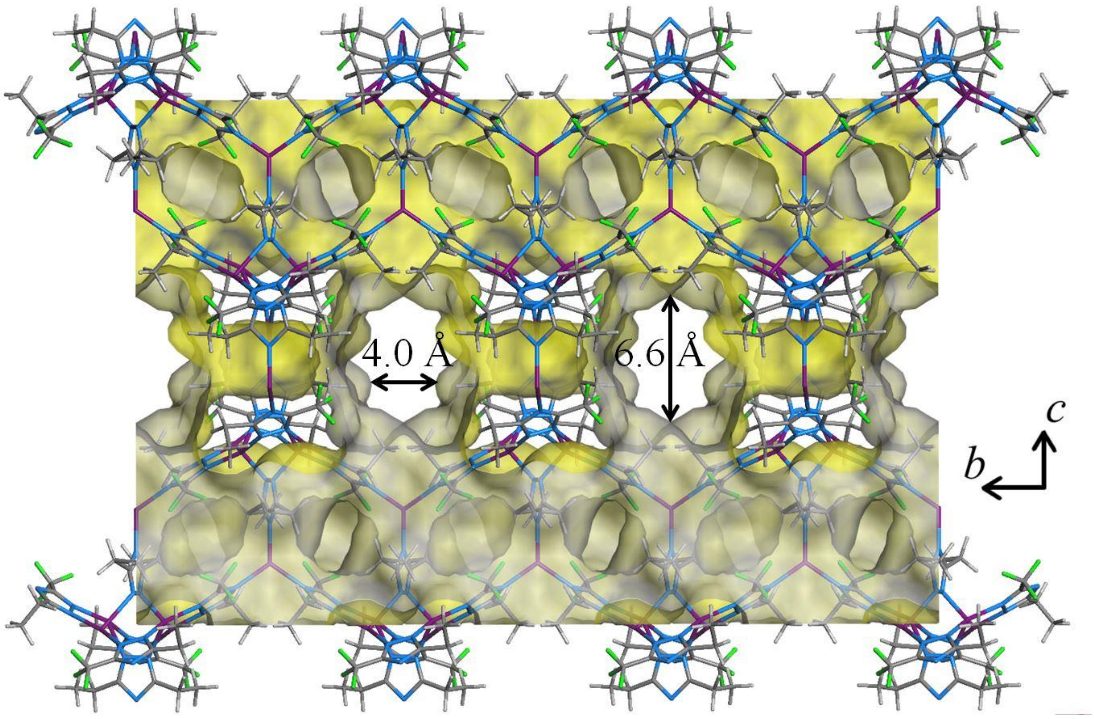

(a)

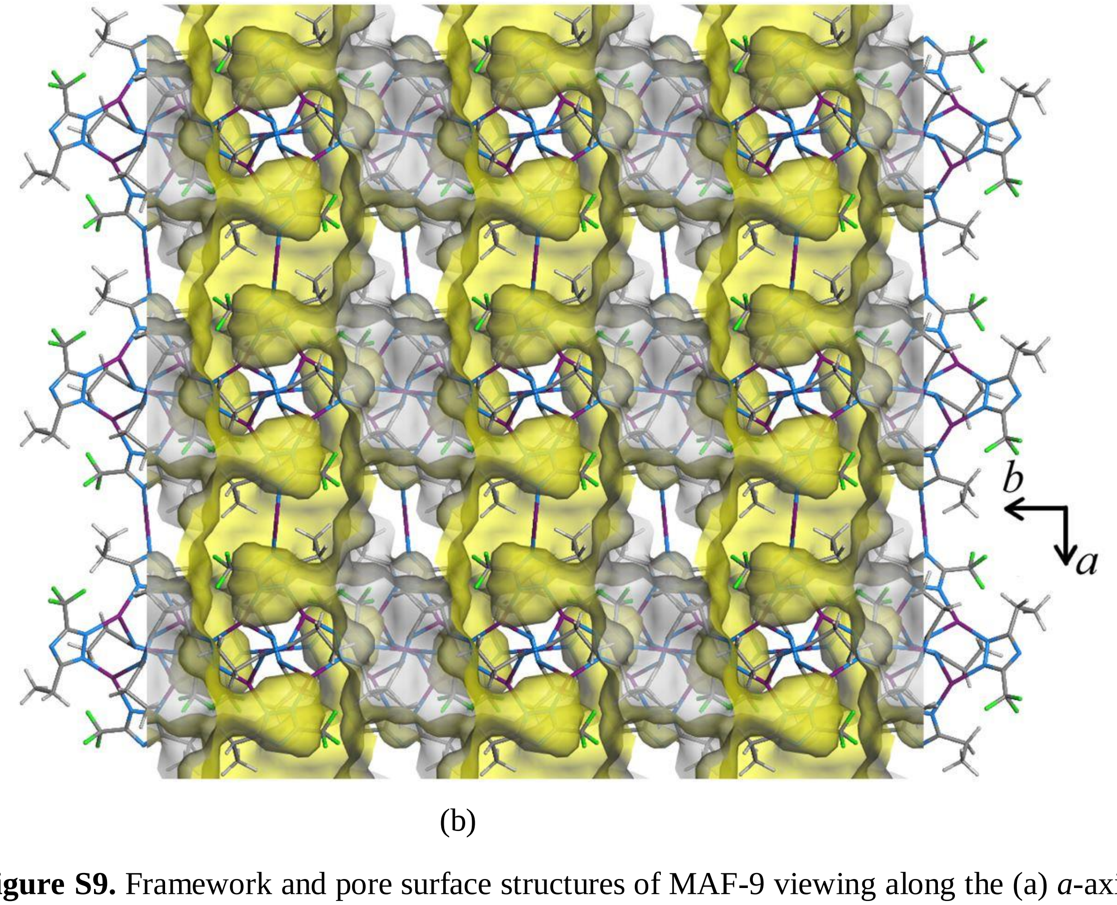

(b)

Figure S9. Framework and pore surface structures of MAF-9 viewing along the (a) *a*-axis and (b) the *c*-axis, supposing that the disordered fetz⁻ ligand contains two $–C_2H_5$ groups. The red circles highlight the connection between the 0D cavities and 3D channel.

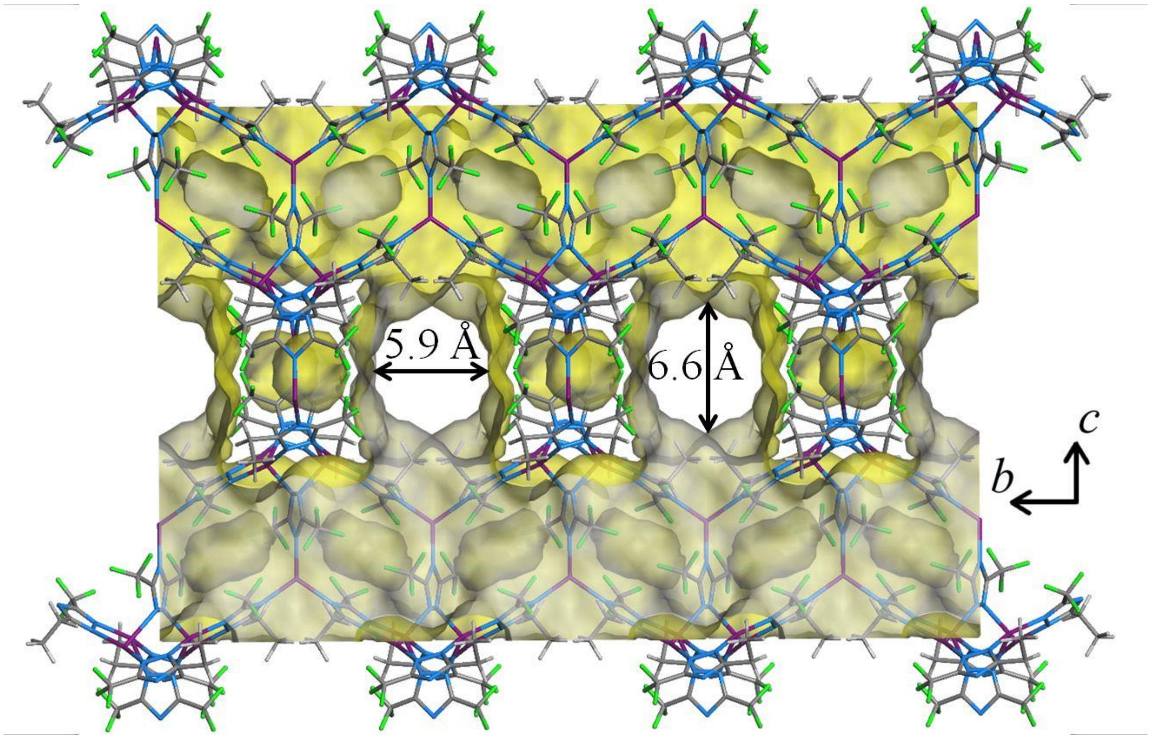

(a)

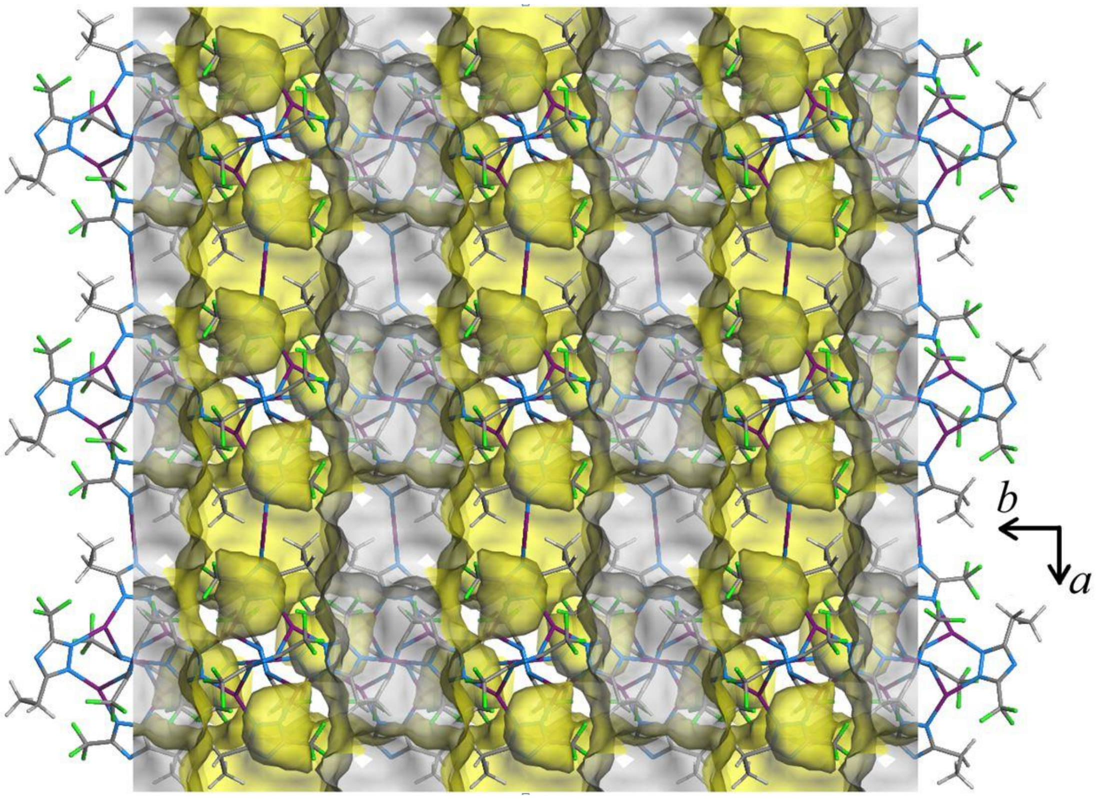

(b)

Figure S10. Framework and pore surface structures of MAF-9 viewing along the (a) *a*-axis and (b) the *c*-axis, supposing that the disordered fetz^{-} ligand contains two $–CF_3$ groups.

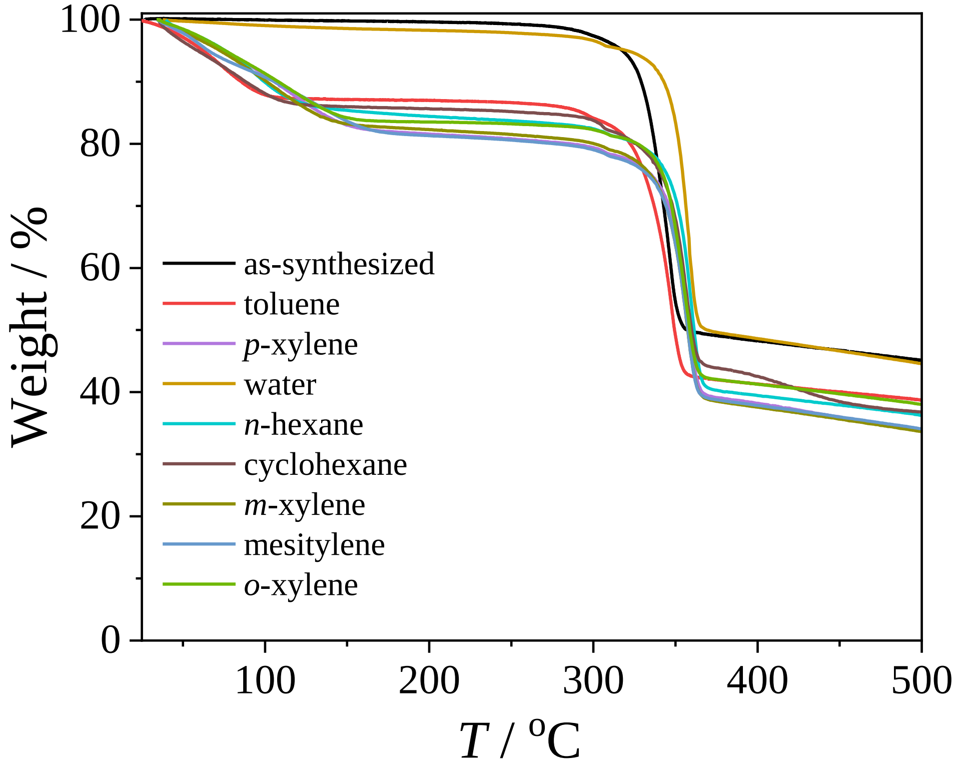

Figure S11. Thermogravimetry curves of MAF-9 after exposed in saturated solvent vapor at room temperature for 3 days.

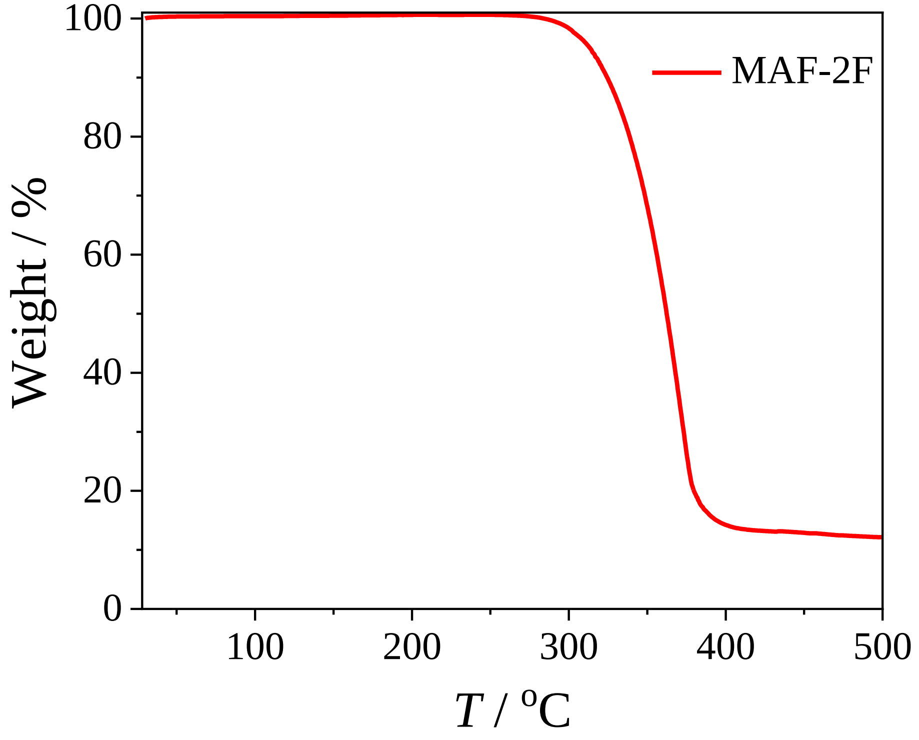

Figure S12. Thermogravimetry curve of MAF-2F.

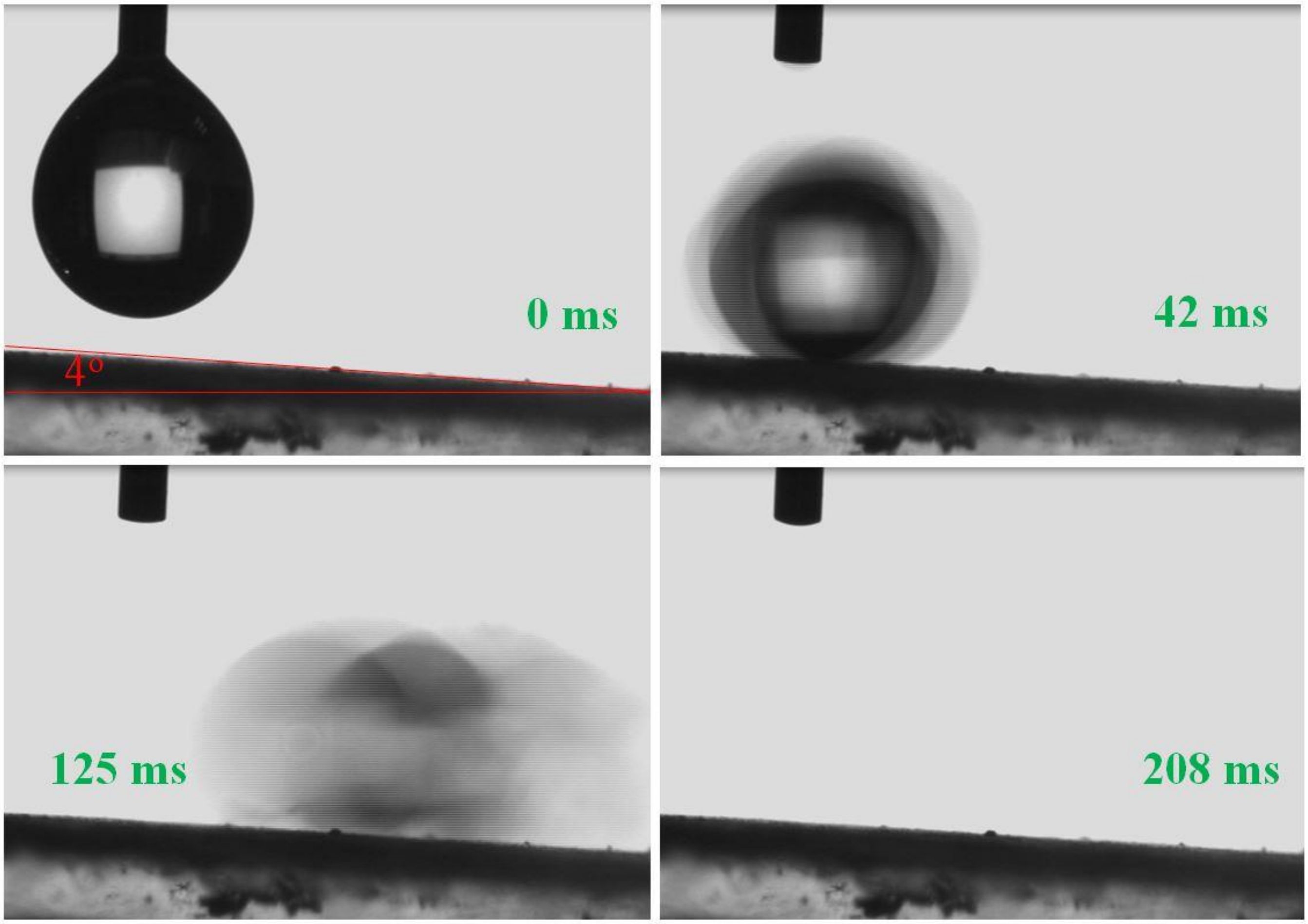

Figure S13. Selected photographs showing the rolling of a water droplet on the compact powder sample of MAF-9 with a glide angle of 3.8°.

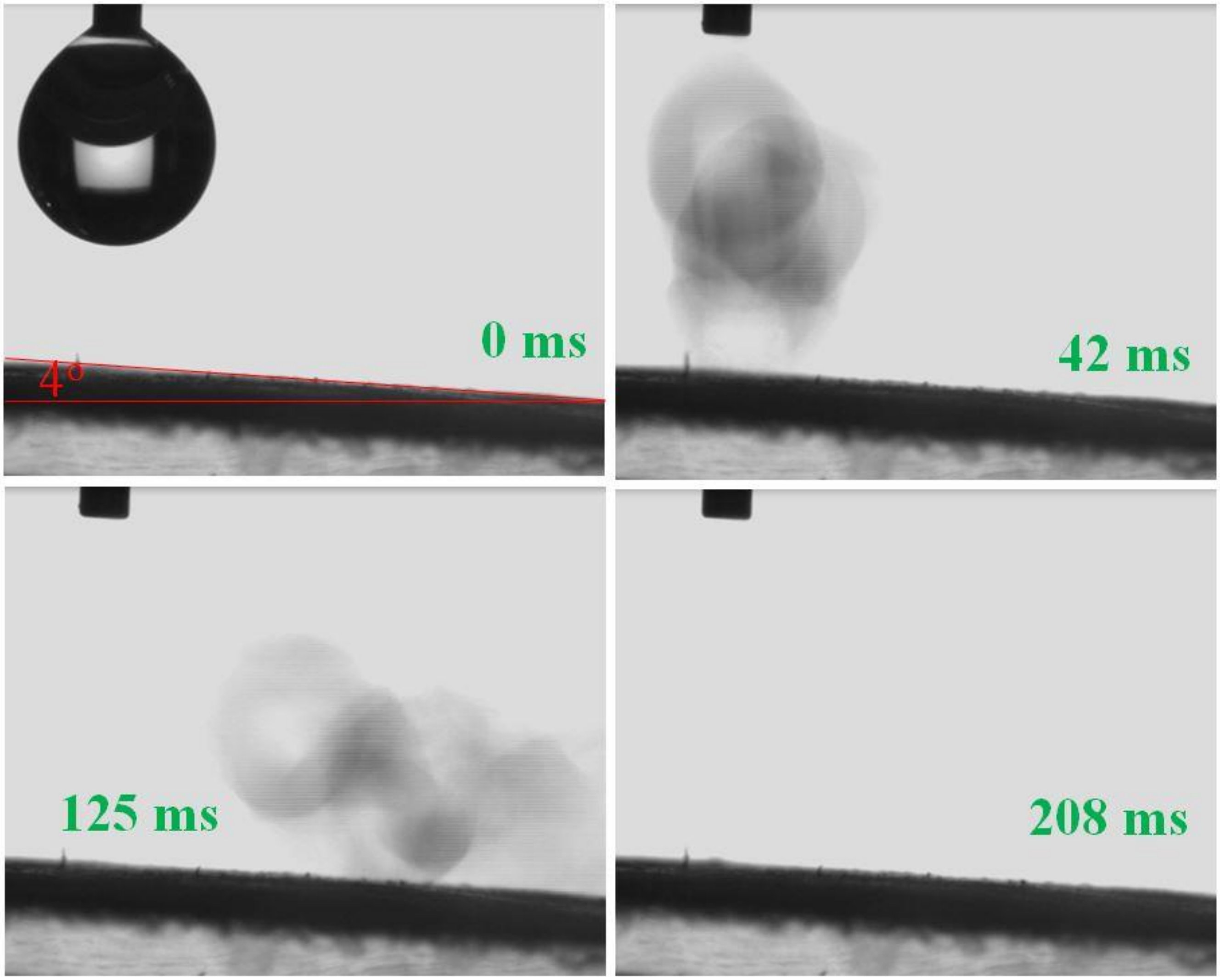

Figure S14. Selected photographs showing the rolling of a water droplet on the compact powder sample of MAF-2F with a glide angle of <4°.

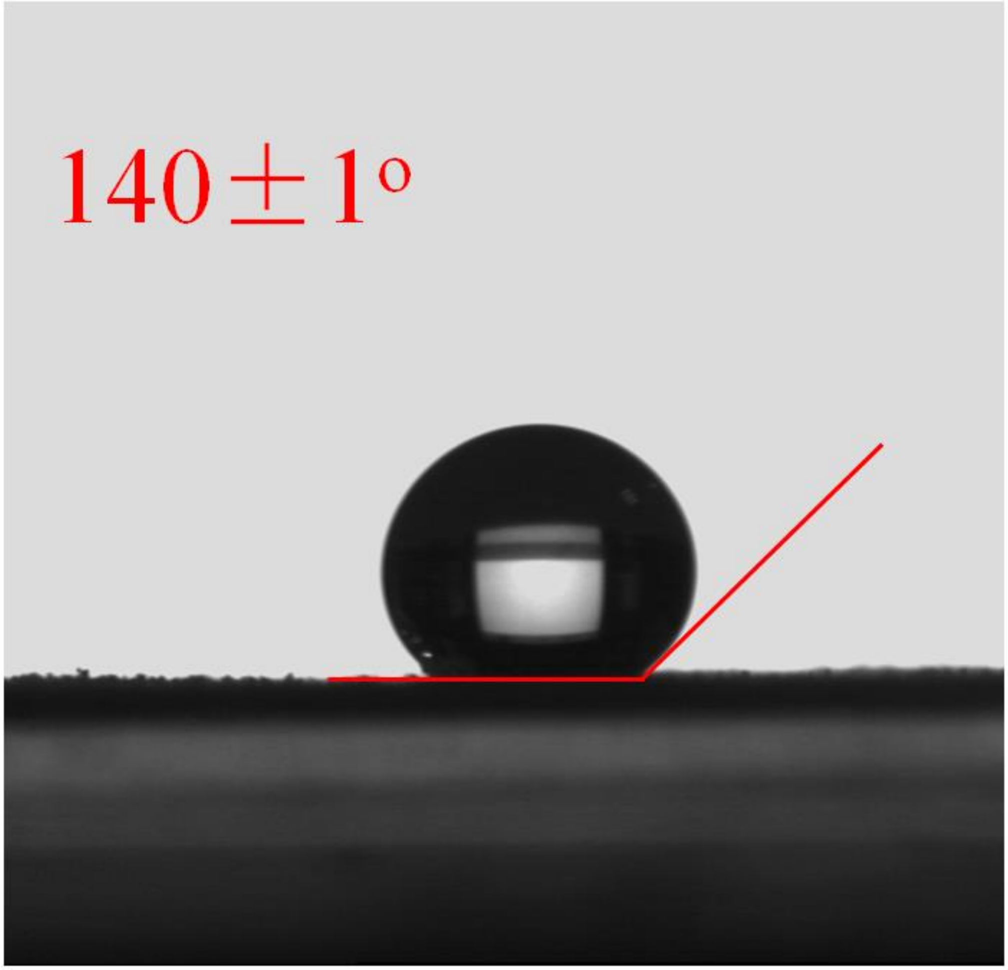

Figure S15. Water contact angle tested for MAF-2.

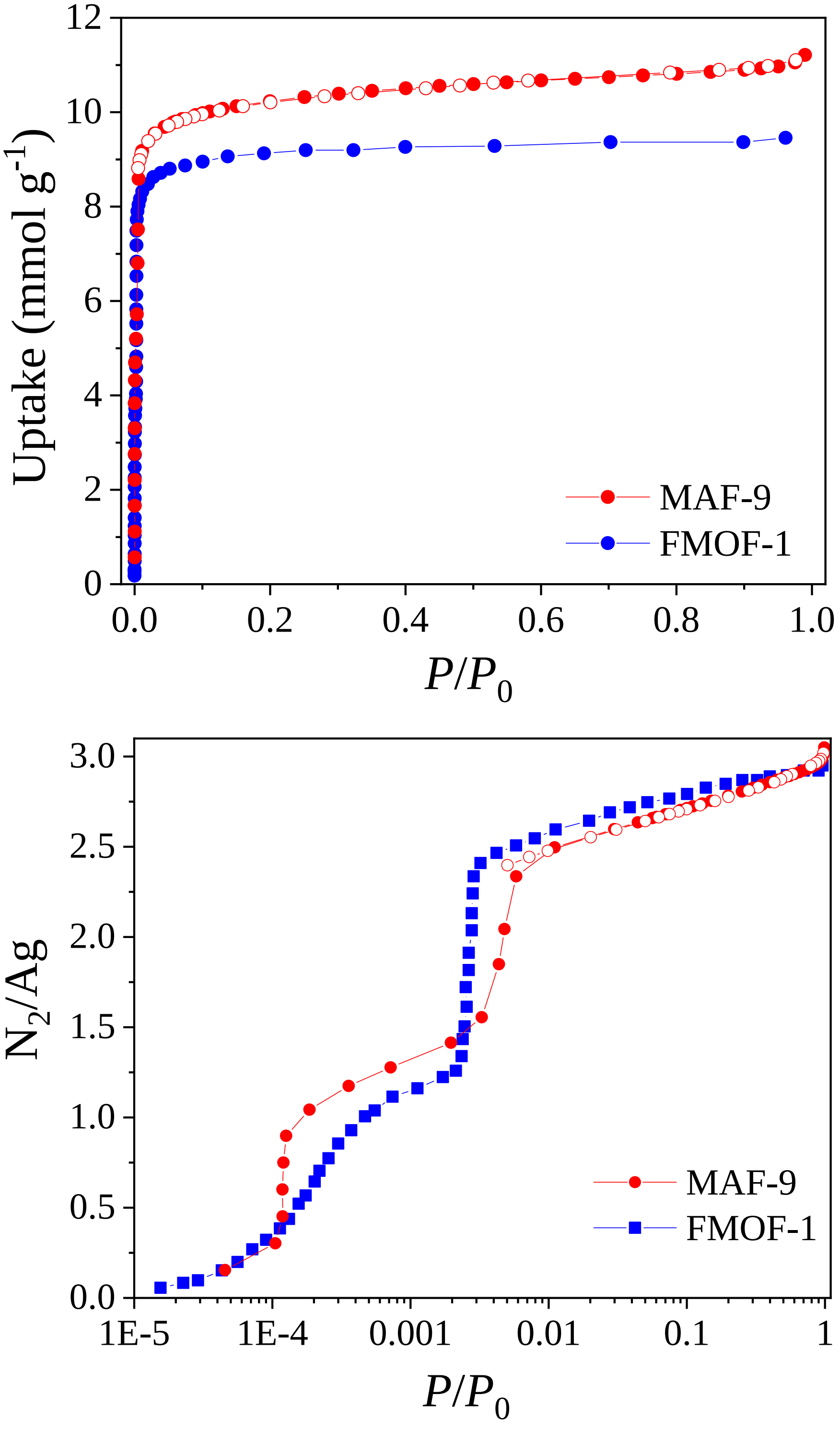

Figure S16. 77 K N_2 adsorption and desorption isotherms of MAF-9 and FMOF-1 presented with different axis settings. Desorption isotherm of FMOF-1 was not reported in the literature.

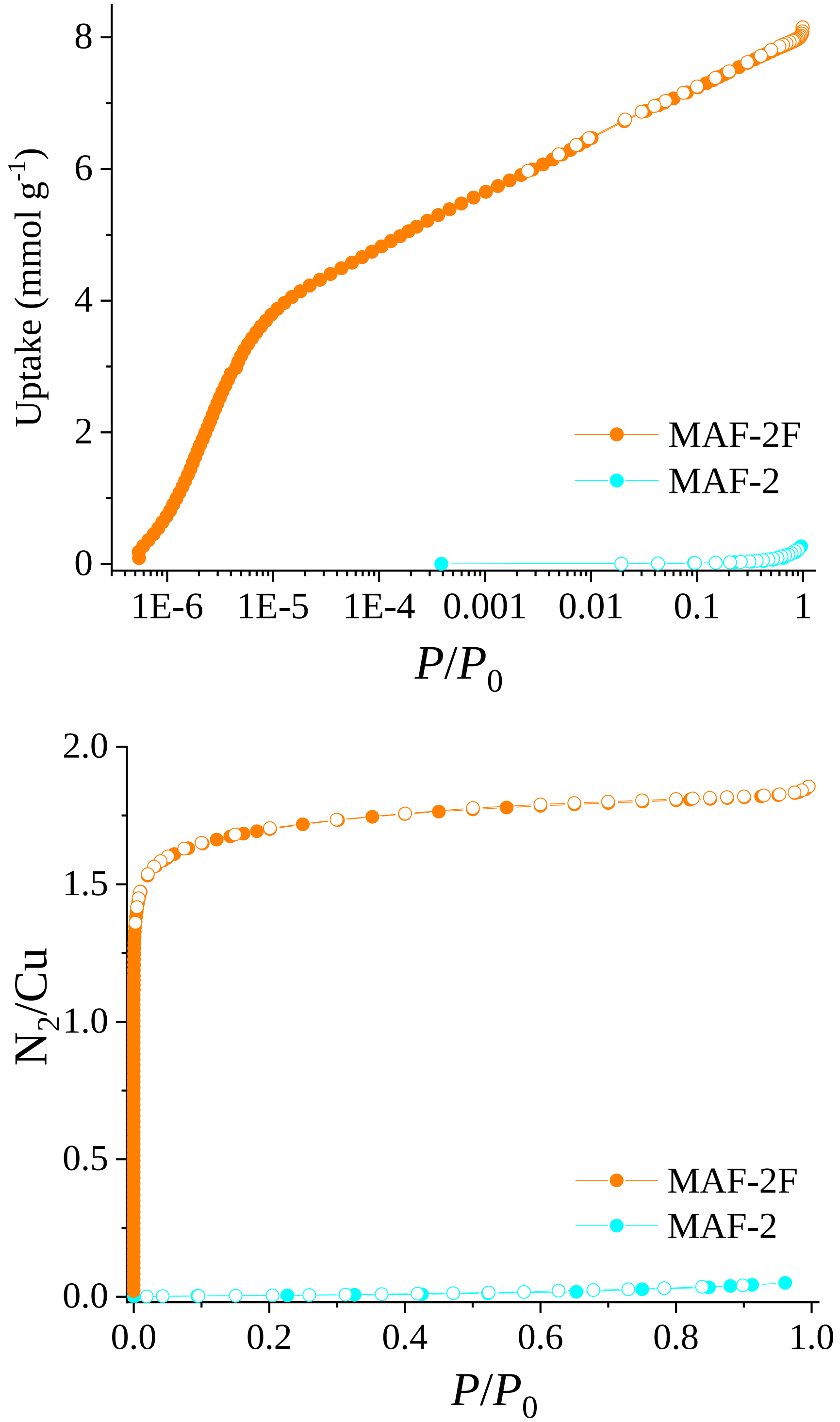

Figure S17. 77 K N_2 adsorption (solid) and desorption (open) isotherms of MAF-2F and MAF-2 presented with different axis settings.

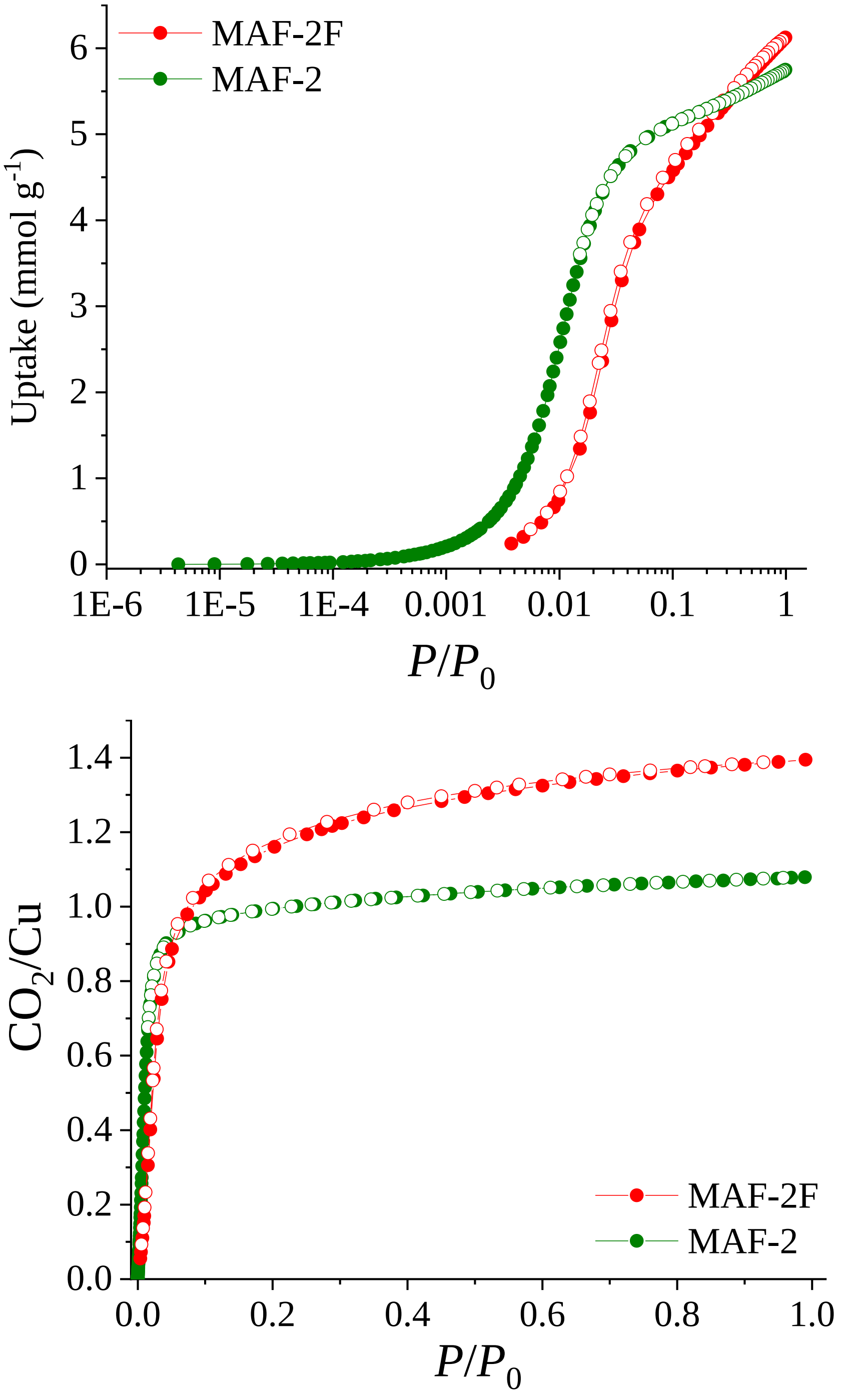

Figure S18. 195 K CO_2 adsorption (solid) and desorption (open) isotherms of MAF-2F and MAF-2 presented with different axis settings.

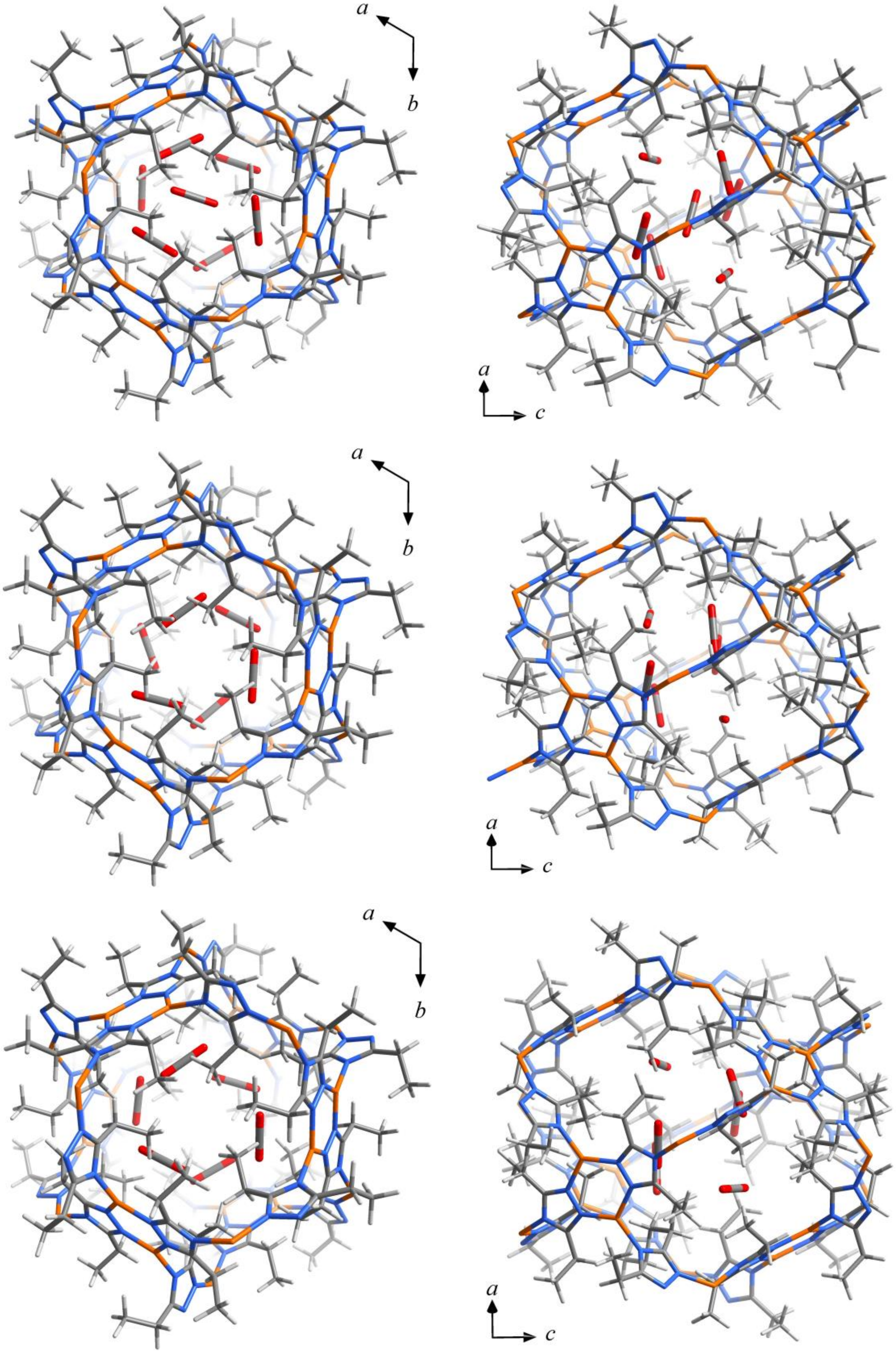

Figure S19. GCMC-derived host-guest structure of MAF-2 saturated with CO_2. From top to bottom are three independent cages in the unit cell, which are projected along the *c*-axis (left) and along the *b*-axis (right). The cages showing 6 CO_2 per cage are structurally very similar with the crystallographic result [J. Am. Chem. Soc. 2009, 131, 5516-5521]. The cage showing 7 CO_2 per cage is different with the crystallographic result mainly for the 1 CO_2 locating at the center of the 6 CO_2. This additional CO_2 appears in only one of the three cages, indicating that the cavity defined by the 6 CO_2 is somewhat repulsive for the 7th CO_2, which is consistent with the relatively large isotherm slope at $P/P_0 > 0.01$ (the slope for common microporous materials is close to zero).

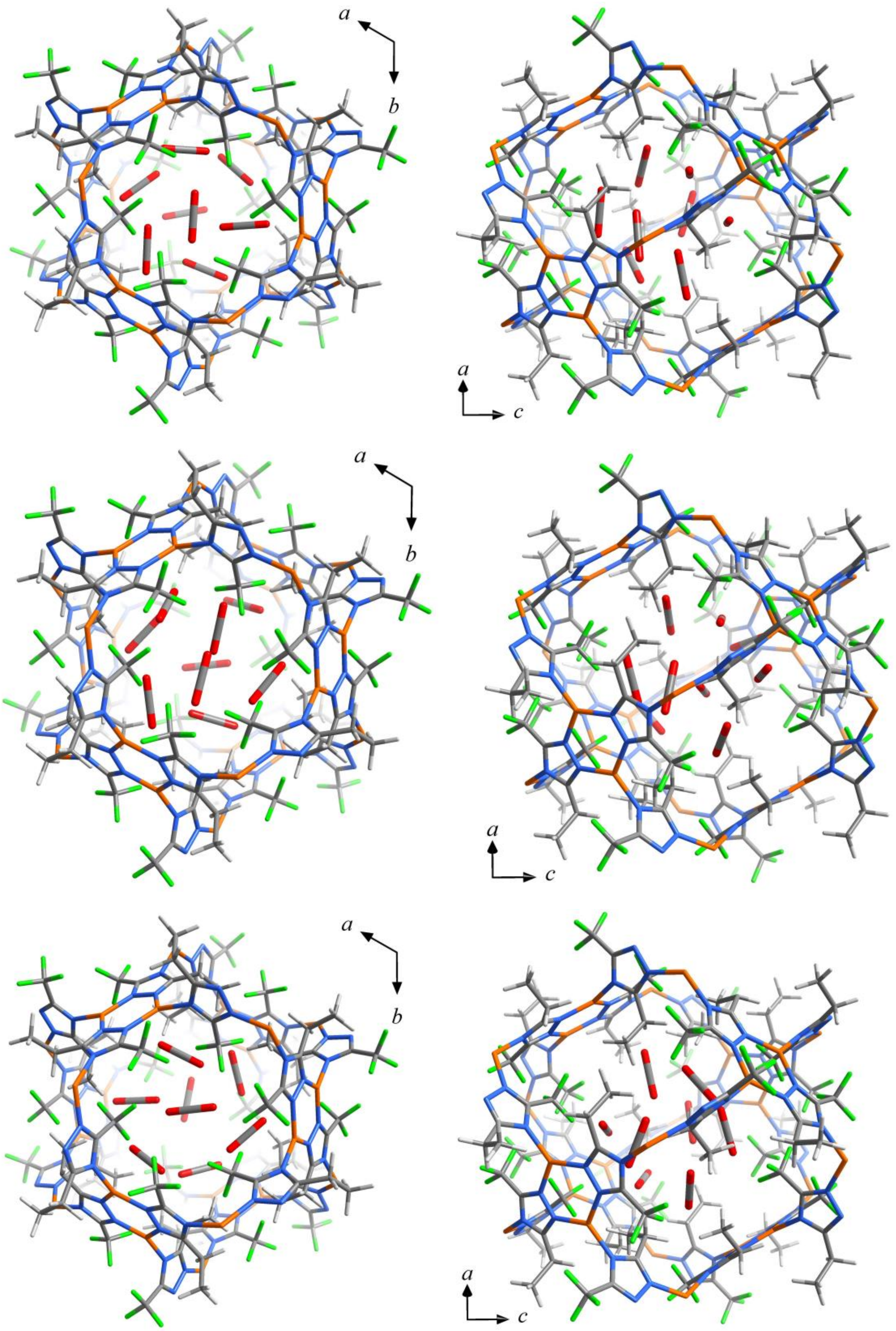

Figure S20. GCMC-derived host-guest structure of MAF-2F saturated with CO_2. From top to bottom are three independent cages in the unit cell, which are projected along the *c*-axis (left) and along the *b*-axis (right). Two cages contain 8 CO_2 molecules per cage, which has 6 CO_2 molecules located around the cage wall, being similar to the crystallographic result of MAF-2. The extra 2 CO_2 molecules locate at the top and bottom of the cages, because the cage is larger than that of MAF-2 mainly at these positions as shown in Figure 3. One cage contains 9 CO_2 molecules, in which the 9th CO_2 locates at the cage bottleneck; meanwhile 1 CO_2 at the cage wall was pushed away. These indicate that the cage of MAF-2F is somewhat too large for accommodating 8 CO_2 molecules and slightly too small for loading 9 CO_2 molecules, being similar with the case of MAF-2.

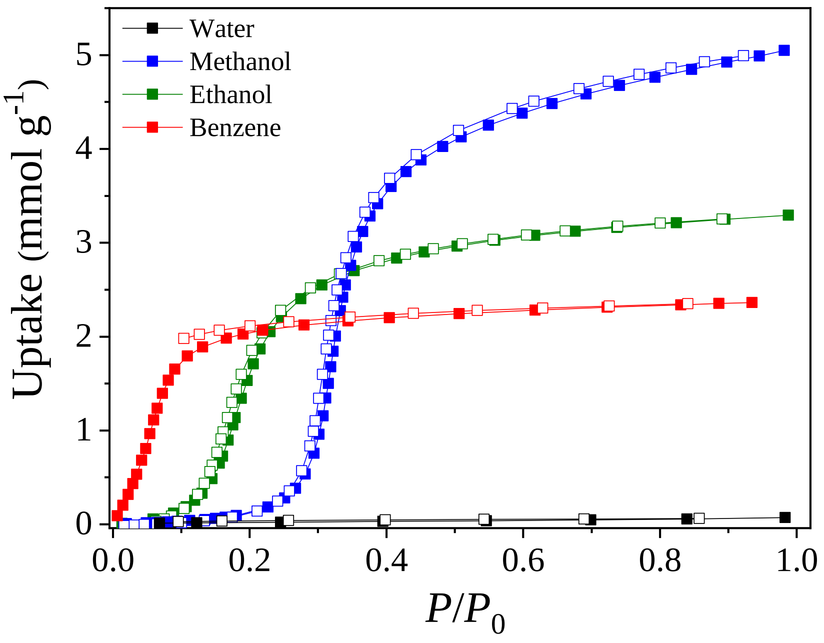

Figure S21. Water, methanol, ethanol, and benzene vapor adsorption (solid) and desorption (open) isotherms of MAF-9 at 298 K.

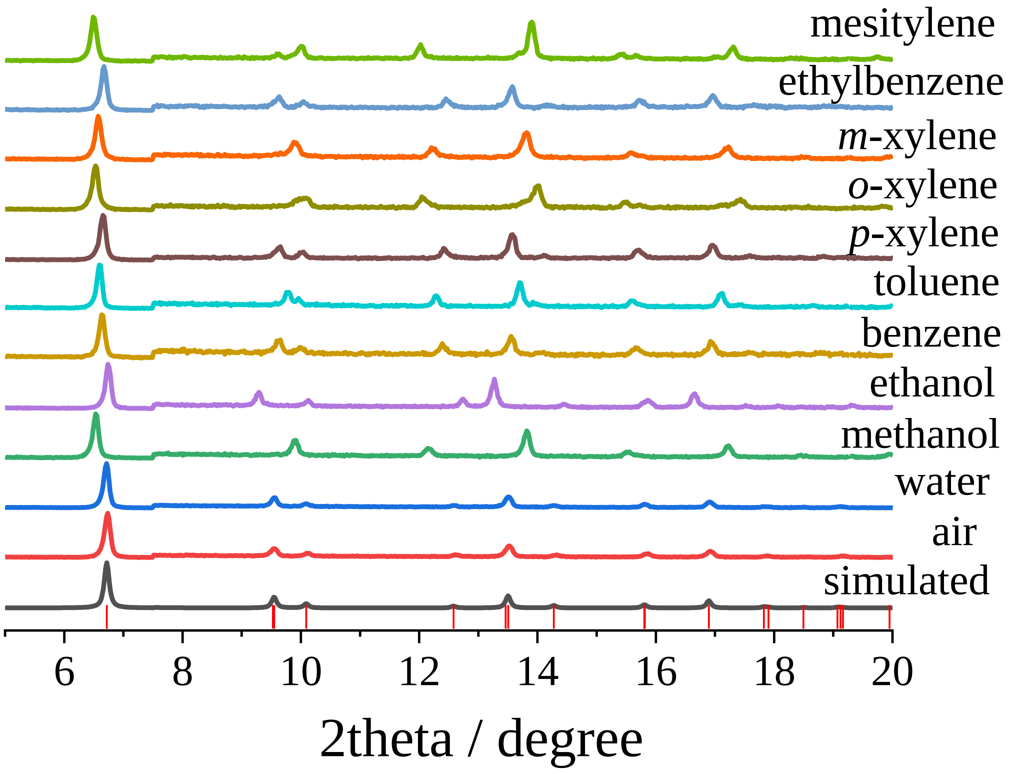

Figure S22. PXRD patterns of MAF-9 in different solvents (measured after immersing for 3 days). For comparison, the data are shown in $2\theta < 20^{o}$, and the diffraction intensity above $2\theta = 7.5^{o}$ are artificially multiplied by 3.

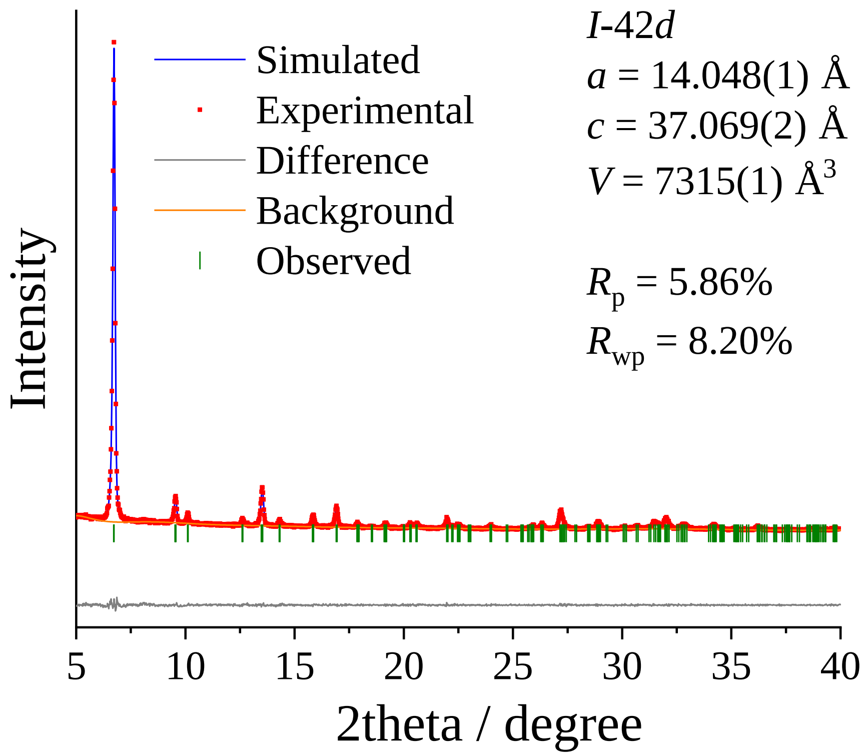

Figure S23. Final Pawley refinement results of the PXRD patterns for MAF-9 in air.

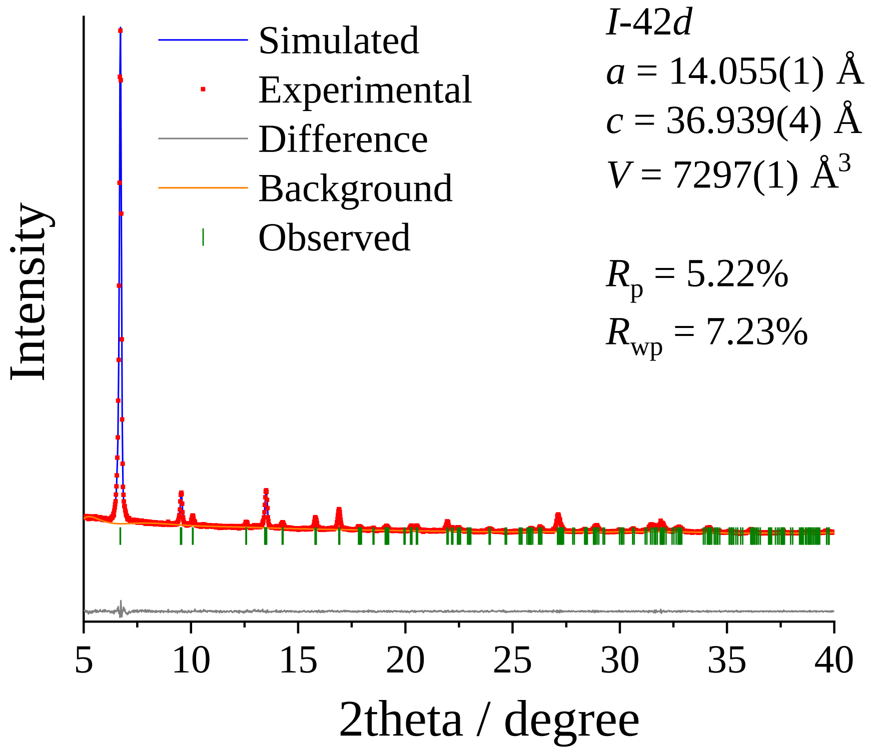

Figure S24. Final Pawley refinement results of the PXRD patterns for MAF-9 in water.

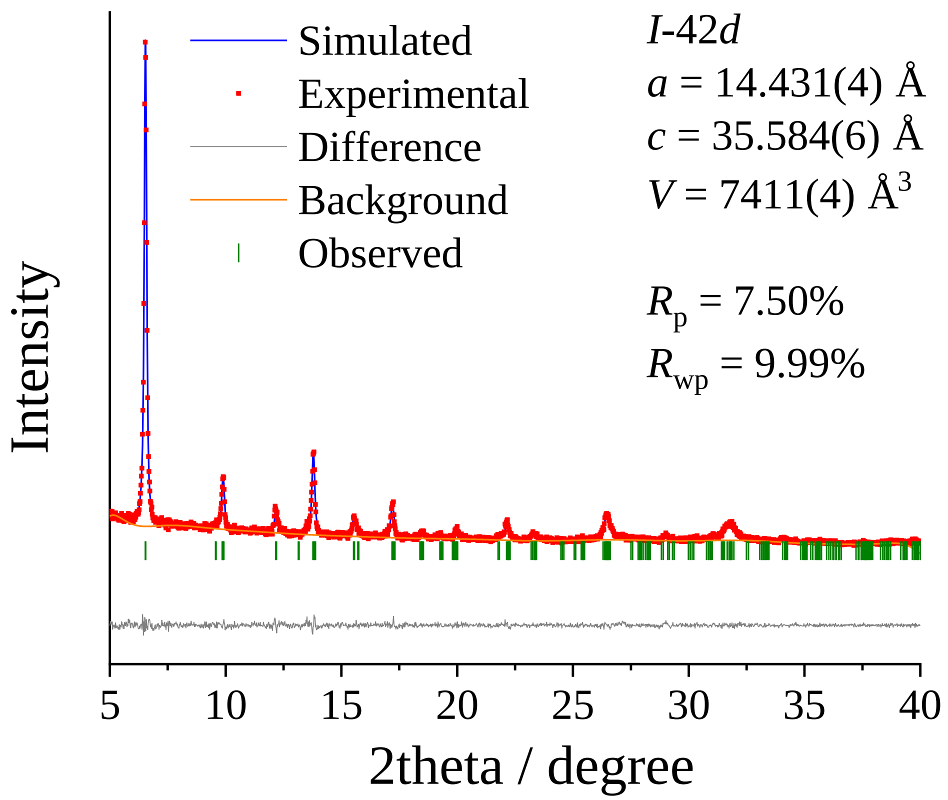

Figure S25. Final Pawley refinement results of the PXRD patterns for MAF-9 in methanol.

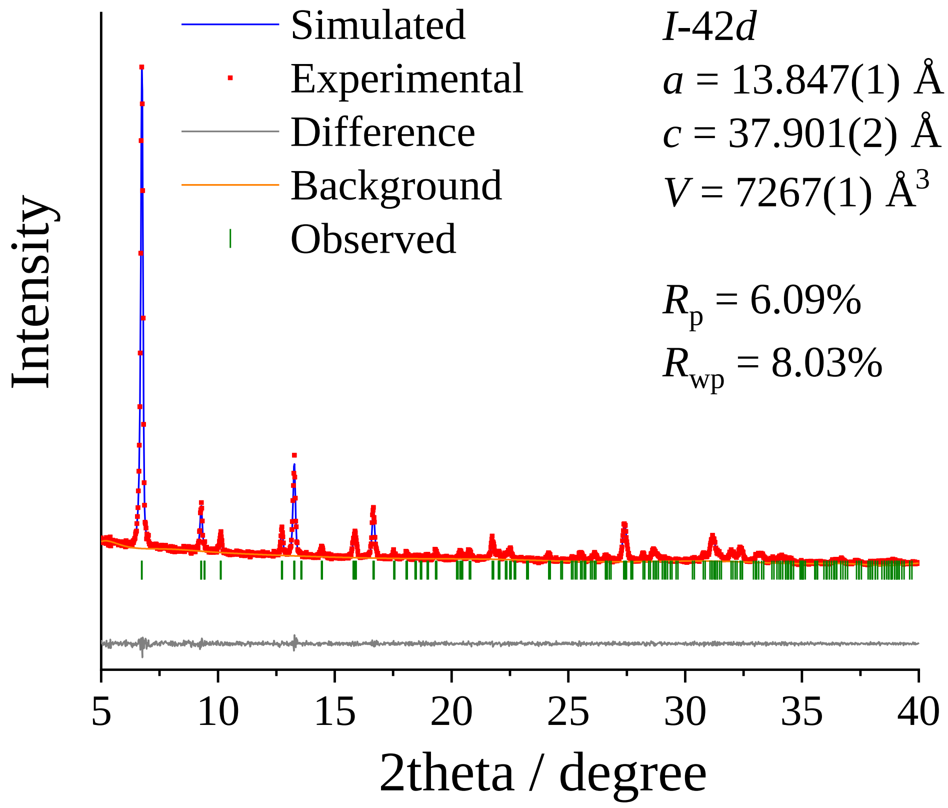

Figure S26. Final Pawley refinement results of the PXRD patterns for MAF-9 in ethanol.

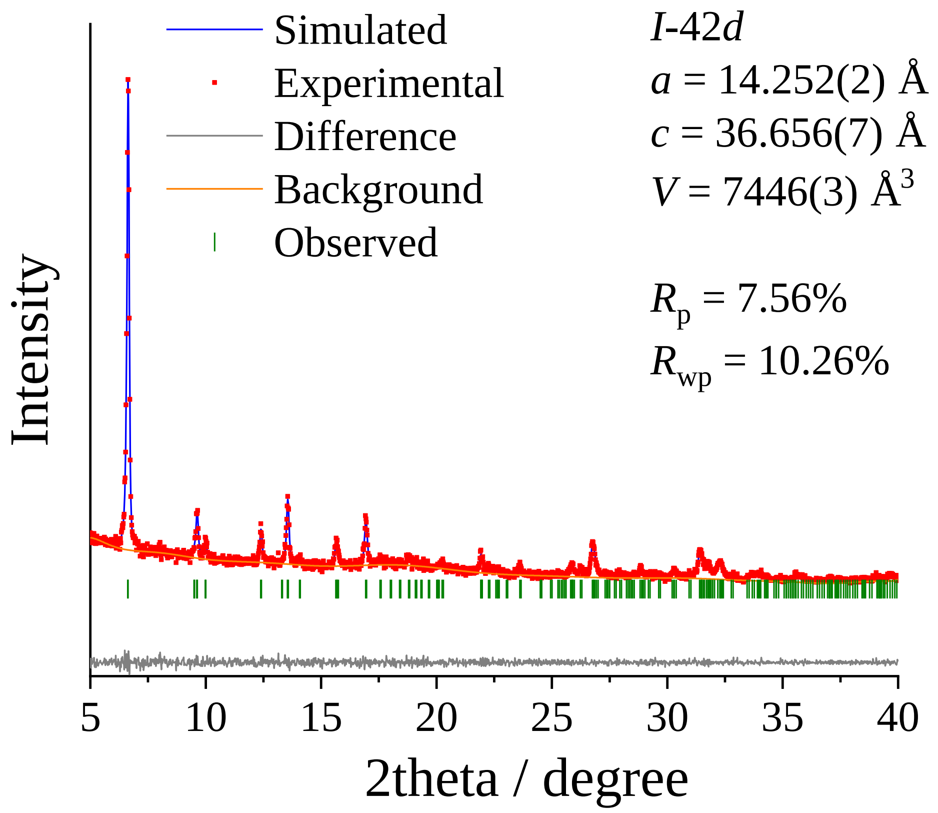

Figure S27. Final Pawley refinement results of the PXRD patterns for MAF-9 in benzene.

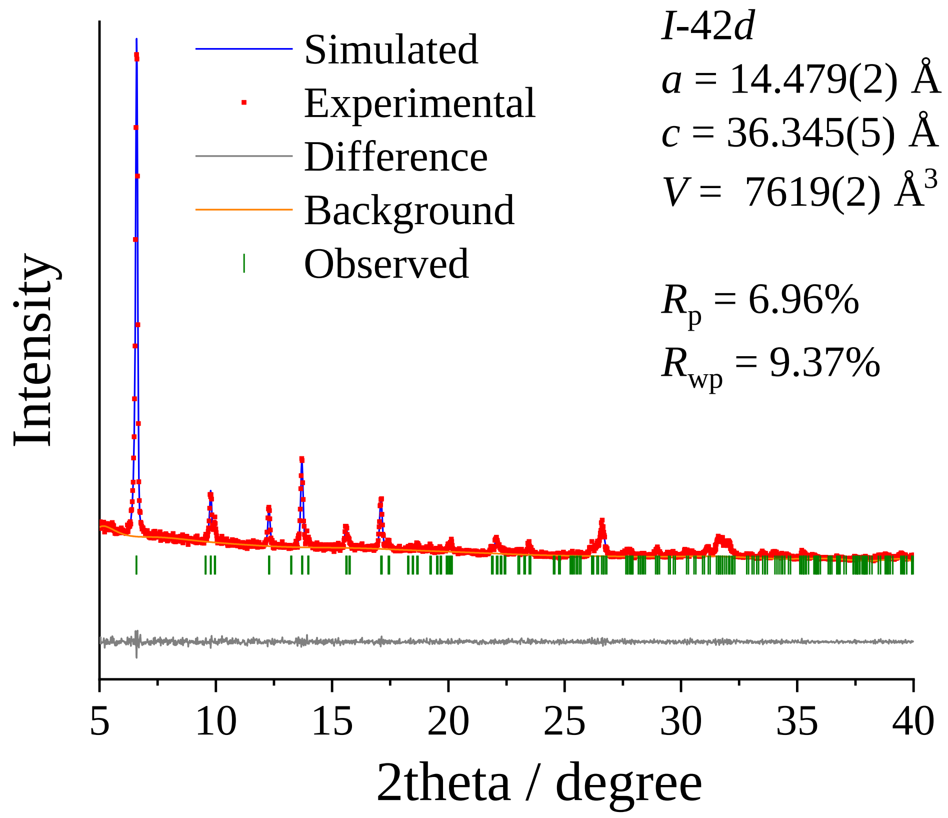

Figure S28. Final Pawley refinement results of the PXRD patterns for MAF-9 in toluene.

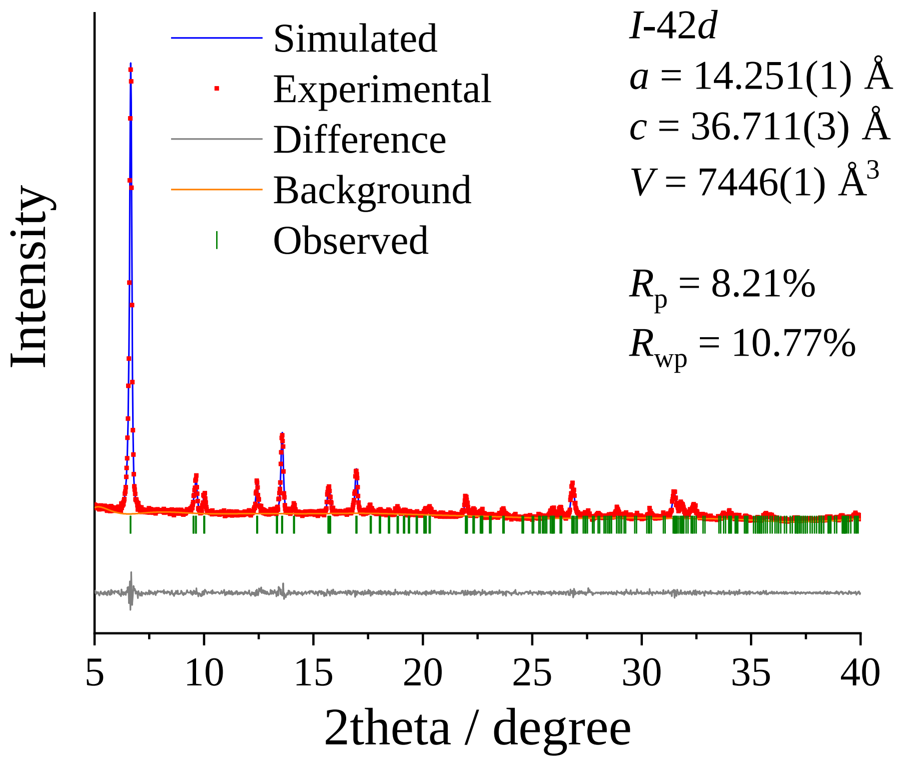

Figure S29. Final Pawley refinement results of the PXRD patterns for MAF-9 in *p*-xylene.

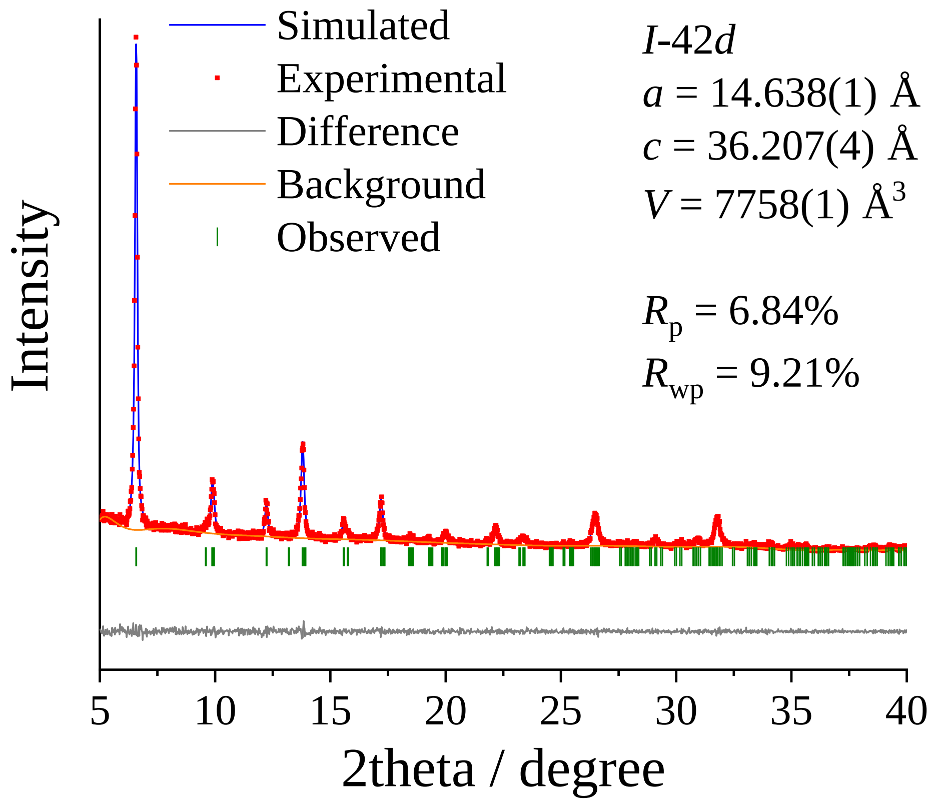

Figure S30. Final Pawley refinement results of the PXRD patterns for MAF-9 in *m*-xylene.

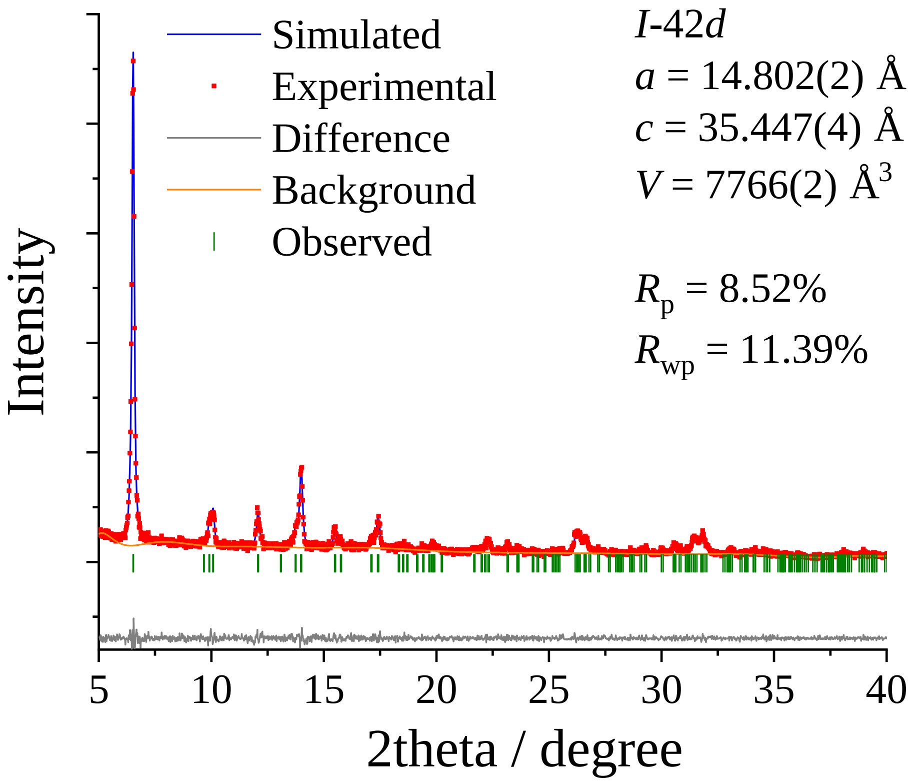

Figure S31. Final Pawley refinement results of the PXRD patterns for MAF-9 in *o*-xylene.

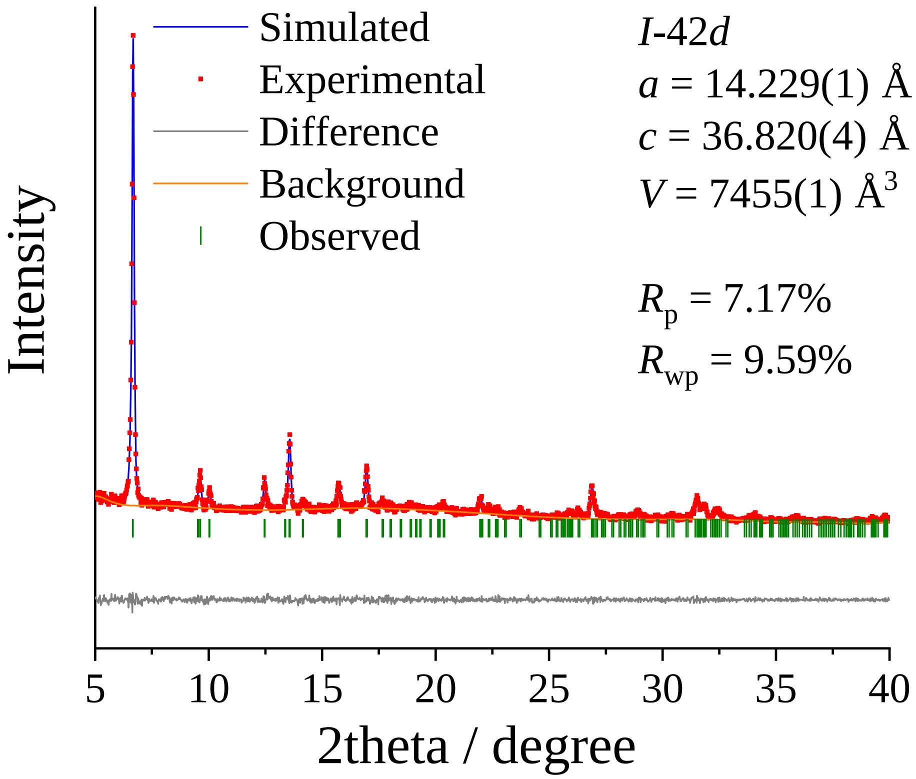

Figure S32. Final Pawley refinement results of the PXRD patterns for MAF-9 in ethylbenzene.

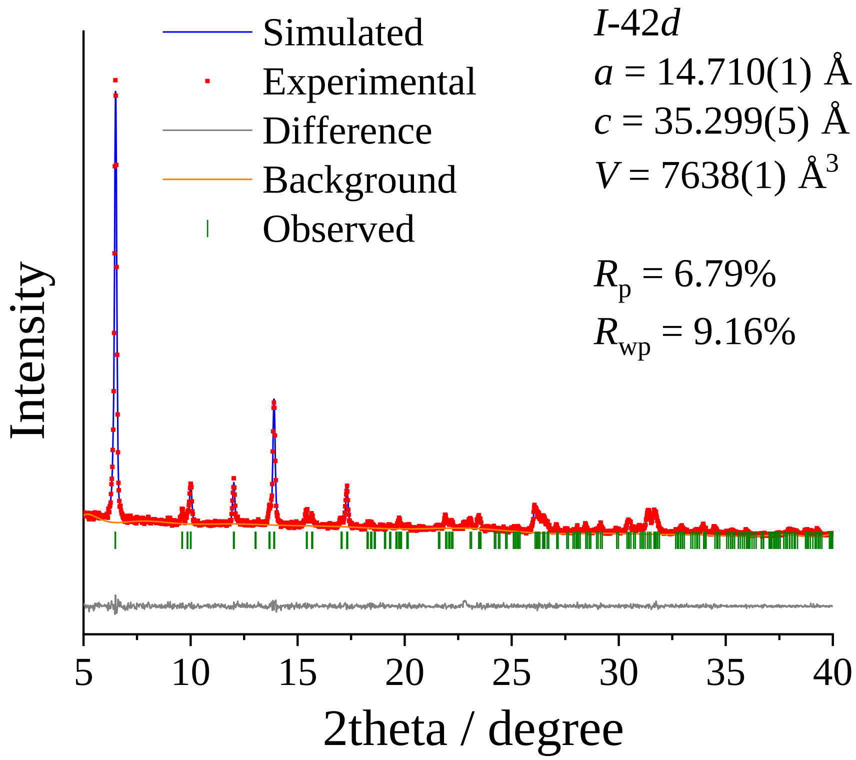

Figure S33. Final Pawley refinement results of the PXRD patterns for MAF-9 in mesitylene.

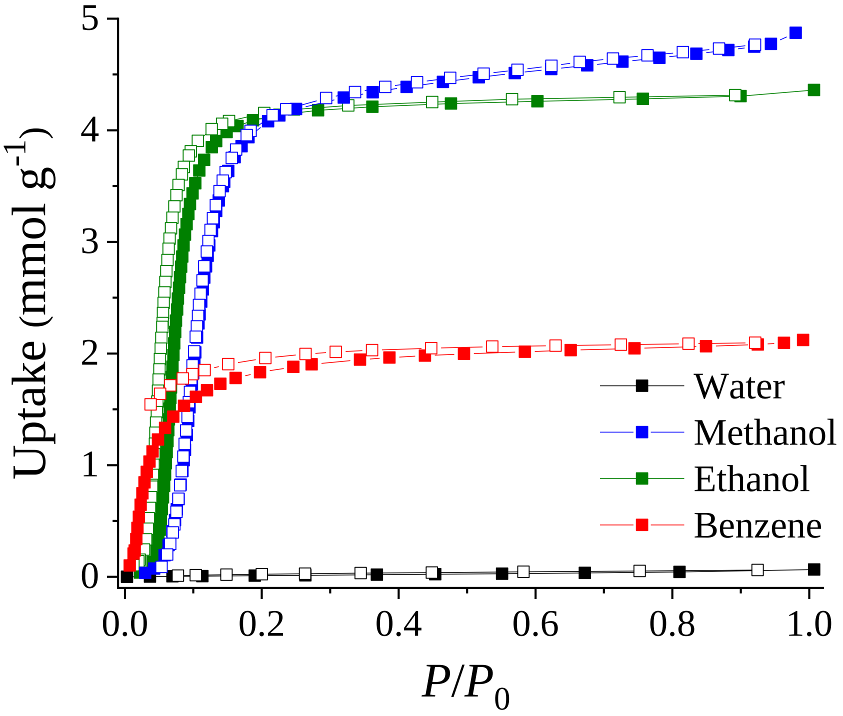

Figure S34. Water, methanol, ethanol, and benzene vapor adsorption (solid) and desorption (open) isotherms of MAF-2F at 298 K.

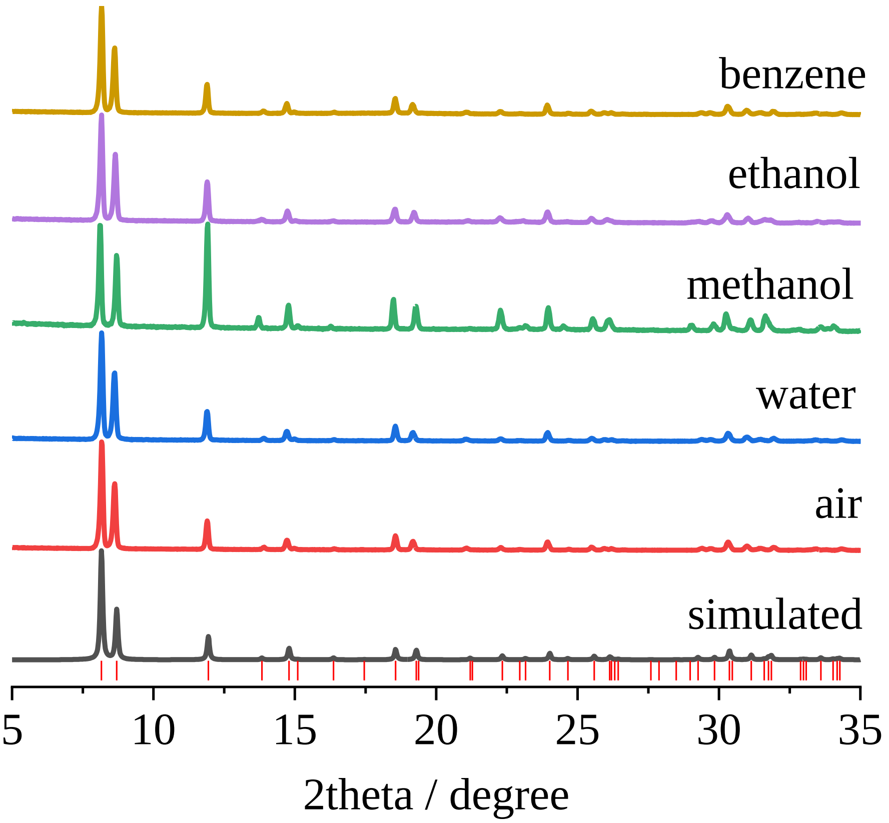

Figure S35. PXRD patterns of MAF-2F in different solvents (measured after immersing for 3 days).

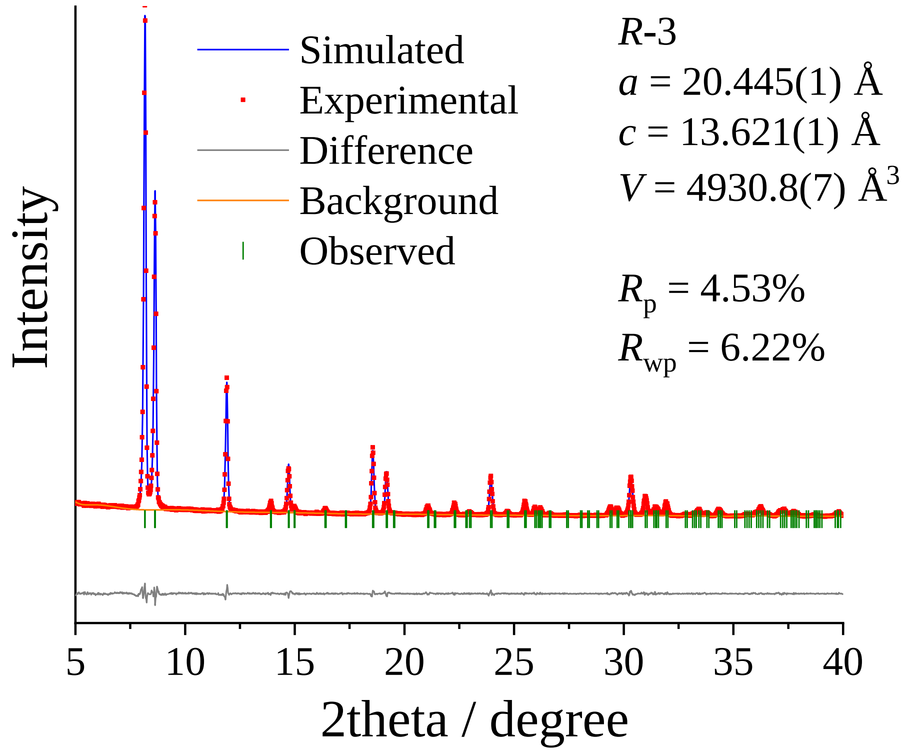

Figure S36. Final Pawley refinement results of the PXRD patterns for MAF-2F in air.

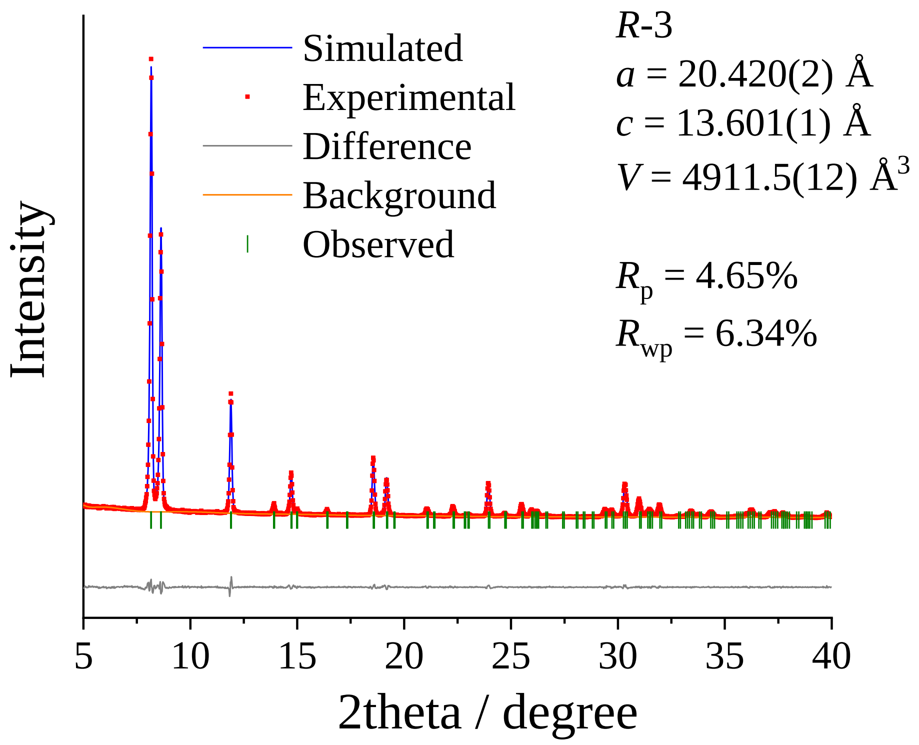

Figure S37. Final Pawley refinement results of the PXRD patterns for MAF-2F in water.

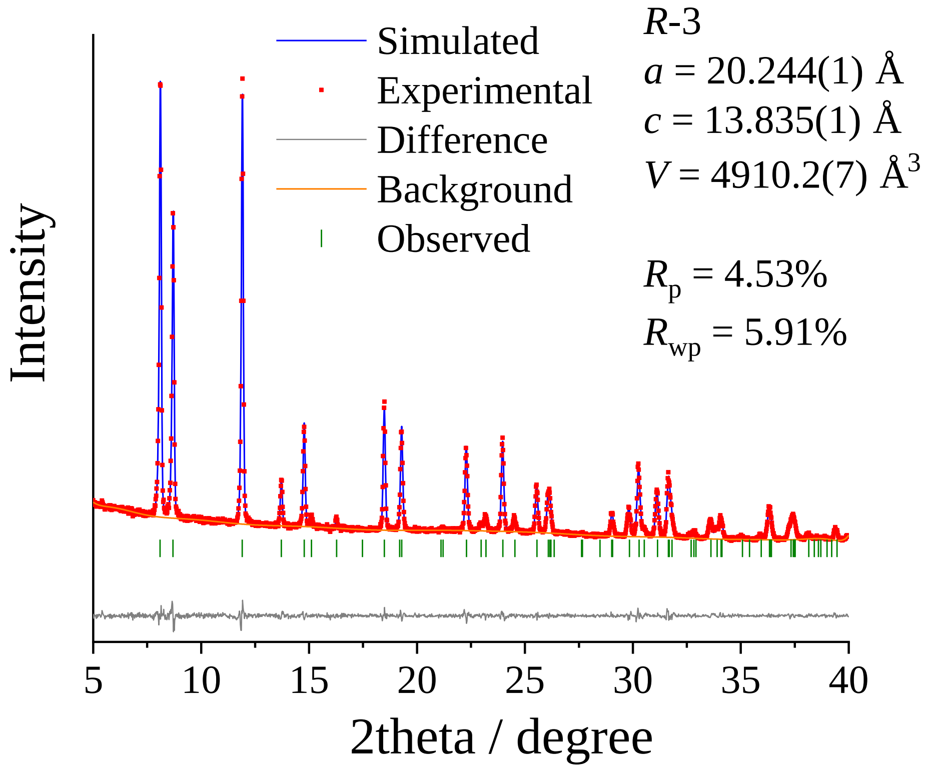

Figure S38. Final Pawley refinement results of the PXRD patterns for MAF-2F in methanol.

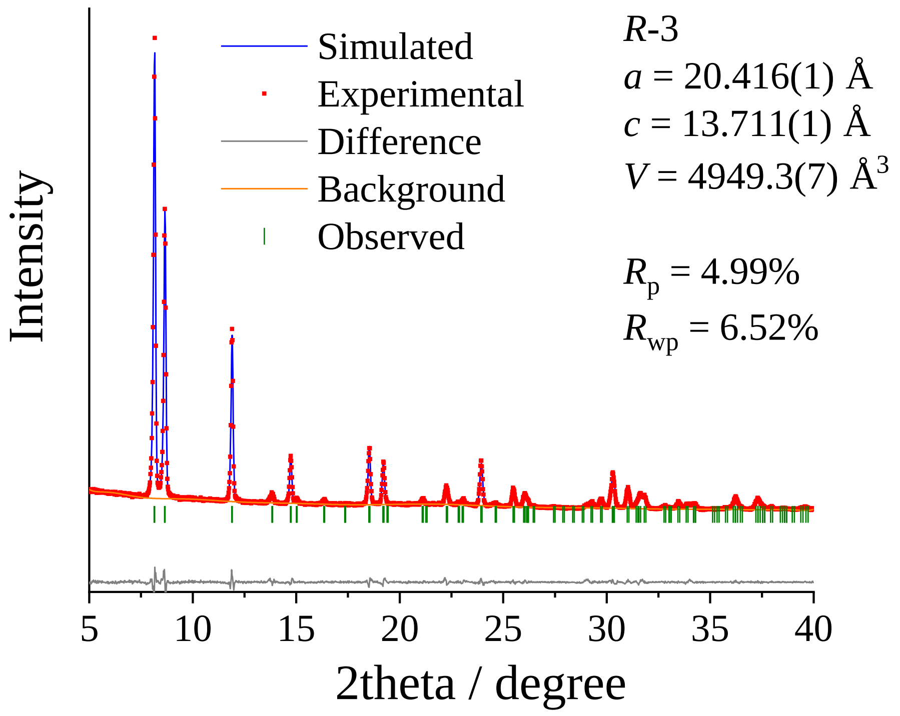

Figure S39. Final Pawley refinement results of the PXRD patterns for MAF-2F in ethanol.

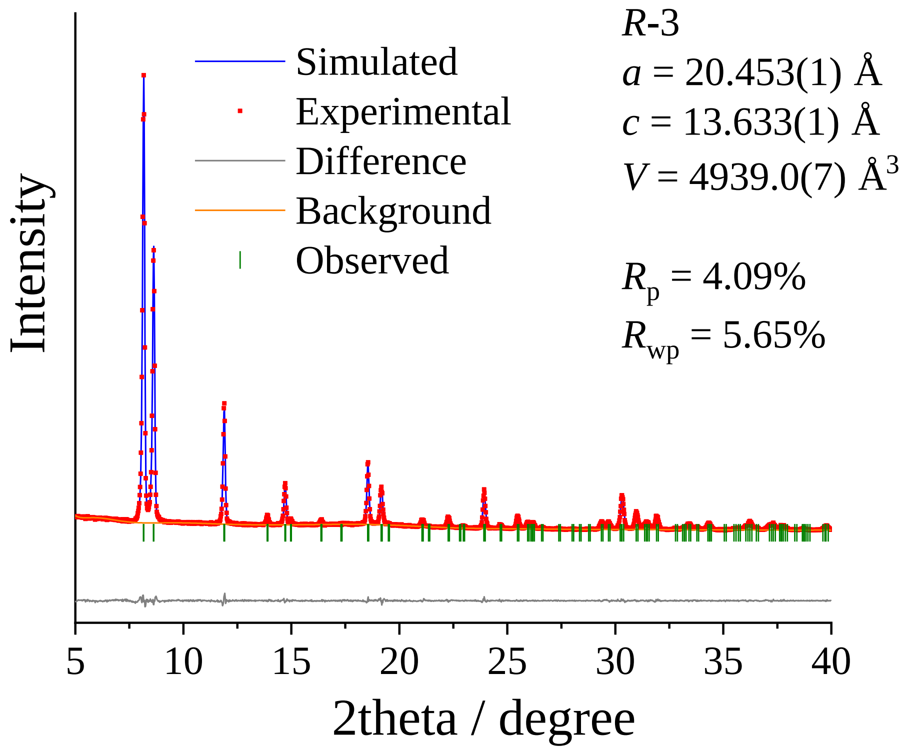

Figure S40. Final Pawley refinement results of the PXRD patterns for MAF-2F in benzene.

Table S1. Crystal data and structure refinement results.

Compound	MAF-9	MAF-9	FMOF-1	MAF-2F	MAF-2
Formula	$AgC_5F_3H_5N_3$	$AgC_5F_3H_5N_3$	$AgC_4F_6N_3$	$C_5CuF_3H_5N_3$	$C_6CuH_{10}N_3$
Formula weight	271.99	271.99	311.94	227.66	187.71
Temperature (K)	173(2)	298(2)	295(2)	298(2)	298(2)
Space group	*I*-42*d*	*I*-42*d*	*I*-42*d*	*R*-3	*R*-3
a (Å)	13.6550(5)	14.0620(12)	14.0733(5)	20.3065(7)	19.6809(3)
b (Å)	13.6550(5)	14.0620(12)	14.0733(5)	20.3065(7)	19.6809(3)
c (Å)	37.990(4)	37.021(4)	37.675(3)	13.7304(5)	14.1877(3)
V (Å^3)	7083.6(8)	7320.6(15)	7461.83	4903.2(3)	4759.18(18)
Z	24	24	24	18	18
D_c (g cm^{-3})	1.530	1.481	1.666	1.388	1.179
R_{int}	0.1012	0.0500	/	0.0342	0.0240
R_1 (>2σ)^a	0.080	0.0850	/	0.0643	0.0464
wR_2 (>2σ)^b	0.1919	0.1461	/	0.1713	0.1453
R_1 (all data)	0.1544	0.1459	/	0.0749	0.0475
wR_2 (all data)	0.2402	0.1753	/	0.1859	0.1470
GOF	0.967	1.031	/	1.093	1.073
Flack	-0.02(3)	0.06(5)	/	/	/

^{a} $R_1 = \sum||F_o| - |F_c||/\sum|F_o|$; ^{b} $wR_2 = [\sum w(F_o^2 - F_c^2)^2/\sum w(F_o^2)^2]^{1/2}$

Table S2. Comparison of the coordination bond lengths (Å) of MAF-9 and FMOF-1.

	MAF-9	FMOF-1
Ag1−N1	2.14(3)	2.245(11)
Ag1−N3, Ag1−N3A	2.25(2)	2.297(8)
Ag2−N2, Ag2−N2B	2.53(3)	2.616(9)
Ag2−N4C	2.14(3)	2.193(8)
Ag2−N5D	2.20(3)	2.194(8)

Symmetry codes: A = –*x*, 1−*y*, *z*; B = 1/2−*y*, 1/2+*x*, 1/2−*z*; C = −1+*y*, 1/2+*x*, 1/4+*z*; D = −*x*, −1/2+*y*, 1/4−z.

Table S3. Comparison of the porosity parameters of MAF-9 and FMOF-1.

Compound	MAF-9	FMOF-1
Formula weight	271.99	311.94
Crystal density (g cm^{-3})	1.481	1.666
Channel size ($Å^2$)	4.0-5.9 × 6.6	5.8 × 7.9
Void ratio	41.0%	44.4%
Crystallographic pore volume (cm^3 g^{-1})	0.277	0.267
N_2 pore volume (cm^3 g^{-1})	0.382	0.328
N_2 saturated uptake (mmol g^{-1})	10.97	9.41
N_2 saturated uptake (mmol cm^{-3})	16.25	15.68
N_2 saturated uptake (N_2/Ag)	2.98	2.94

Table S4. Comparison of the coordination bond lengths (Å) of MAF-2F and MAF-2.

	MAF-2F	MAF-2
Cu1−N1	1.978(4)	1.983(2)
Cu1−N2B	1.961(3)	1.950(2)
Cu1−N3C	1.990(3)	1.974(2)

Symmetry codes: B = 1−*x*, 1−*y*, 1−*z*, C = −1/3+*y*, 1/3−*x*+*y*, 4/3−*z*.

Table S5. Comparison of the porosity parameters of MAF-2F and MAF-2.

Compound	MAF-2F	MAF-2
Formula weight	227.66	187.71
Crystal density (g cm^{-3})	1.388	1.179
Cavity/Aperture diameter (Å)	7.8~11.0/2.5	7.2~11.0/1.1
Void ratio	38.3%	31.3%
Crystallographic pore volume (cm^3 g^{-1})	0.276	0.265
N_2/CO_2 pore volume (cm^3 g^{-1})	0.279/0.243	0.008/0.228
N_2 saturated uptake (mmol g^{-1})	8.00	0.23
N_2 saturated uptake (mmol cm^{-3})	11.10	0.27
N_2 saturated uptake (N_2/Cu)	1.82	0.04
CO_2 saturated uptake (mmol g^{-1})	6.10	5.73
CO_2 saturated uptake (mmol cm^{-3})	8.47	6.76
CO_2 saturated uptake (CO_2/Cu)	1.39	1.08

Table S6. PXRD derived unit-cell parameters of MAF-9 in different solvents.

Solvent	Air	Water	Methanol	Ethanol	Benzene	Toluene
a (Å)	14.048(1)	14.055(1)	14.431(4)	13.847(1)	14.252(2)	14.479(2)
$\Delta a/a_{Air}$	NA	+0.05%	+2.7%	–1.4%	+1.5%	+3.1%
c (Å)	37.069(2)	36.939(4)	35.584(6)	37.901(2)	36.656(7)	36.345(5)
$\Delta c/c_{Air}$	NA	–0.35%	–4.0%	+2.2%	–1.1%	–2.0%
V (Å^3)	7315(1)	7297(1)	7411(4)	7267(1)	7446(3)	7619(2)
$\Delta V/V_{Air}$	NA	–0.3%	+1.3%	–0.7%	+1.7%	+4.1%
R_p	0.0586	0.0522	0.0750	0.0609	0.0756	0.0696
R_{wp}	0.0820	0.0723	0.0999	0.0803	0.1026	0.0937

Solvent	*p*-Xylene	*m*-Xylene	*o*-Xylene	Ethylbenzene	Mesitylene
a (Å)	14.251(1)	14.638(1)	14.802(2)	14.229(1)	14.710(1)
$\Delta a/a_{Air}$	+1.4%	+4.2%	+5.4%	+1.3%	+4.7%
c (Å)	36.711(3)	36.207(4)	35.447(4)	36.820(4)	35.299(5)
$\Delta c/c_{Air}$	–1.0%	–2.3%	–4.4%	–0.7%	–4.8%
V (Å^3)	7446(1)	7758(1)	7766(2)	7455(1)	7638(1)
$\Delta V/V_{Air}$	+1.8%	+6.1%	+6.2%	+1.9%	+4.4%
R_p	0.0821	0.0684	0.0852	0.0717	0.0679
R_{wp}	0.1077	0.0912	0.1139	0.0959	0.0916

Table S7. PXRD derived unit-cell parameters of MAF-2F in different solvents.

Solvent	Air	Water	Methanol	Ethanol	Benzene
a (Å)	20.445(1)	20.420(2)	20.244(1)	20.416(1)	20.453(1)
$\Delta a/a_{Air}$	NA	–0.1%	–1.0%	–0.1%	+0.04%
c (Å)	13.621(1)	13.601(1)	13.835(1)	13.711(1)	13.633(1)
$\Delta c/c_{Air}$	NA	–0.1%	+1.5%	+0.7%	+0.09%
V (Å^3)	4930.8(7)	4911.5(12)	4910.2(7)	4949.3(7)	4939.0(7)
$\Delta V/V_{Air}$	NA	–0.4%	–0.5%	+0.4%	+0.2%
R_p	0.0453	0.0465	0.0453	0.0499	0.0409
R_{wp}	0.0622	0.0634	0.0591	0.0652	0.0565
